# The fraction of cancer attributable to modifiable risk factors in England, Wales, Scotland, Northern Ireland, and the United Kingdom in 2015

**DOI:** 10.1038/s41416-018-0029-6

**Published:** 2018-03-23

**Authors:** Katrina F. Brown, Harriet Rumgay, Casey Dunlop, Margaret Ryan, Frances Quartly, Alison Cox, Andrew Deas, Lucy Elliss-Brookes, Anna Gavin, Luke Hounsome, Dyfed Huws, Nick Ormiston-Smith, Jon Shelton, Ceri White, D. Max Parkin

**Affiliations:** 1 Policy and Information Directorate, Cancer Research UK, The Angel Building, 407 St John Street, London, EC1V 4AD UK; 2 NHS National Services Scotland, Information Services Division, Meridian Court, 5 Cadogan Street, Glasgow, G2 6QE Scotland; 3 National Cancer Registration and Analysis Service, Public Health England, 2nd Floor, Skipton House, 80 London Road, London, SE1 6LH UK; 4 Northern Ireland Cancer Registry, Centre for Public Health, Queens University Belfast, Mulhouse Building, Grosvenor Road, Belfast, BT12 6DP Ireland; 5 Welsh Cancer Intelligence and Surveillance Unit, Floor 5, Public Health Wales, Number 2 Capital Quarter, Tyndall Street, Cardiff, CF10 4BZ Wales; 6 Centre for Cancer Prevention, Wolfson Institute of Preventive Medicine, Queen Mary University of London, Charterhouse Square, London, EC1M 6BQ UK

**Keywords:** Risk factors, Cancer epidemiology

## Abstract

**Background:**

Changing population-level exposure to modifiable risk factors is a key driver of changing cancer incidence. Understanding these changes is therefore vital when prioritising risk-reduction policies, in order to have the biggest impact on reducing cancer incidence. UK figures on the number of risk factor-attributable cancers are updated here to reflect changing behaviour as assessed in representative national surveys, and new epidemiological evidence. Figures are also presented by UK constituent country because prevalence of risk factor exposure varies between them.

**Methods:**

Population attributable fractions (PAFs) were calculated for combinations of risk factor and cancer type with sufficient/convincing evidence of a causal association. Relative risks (RRs) were drawn from meta-analyses of cohort studies where possible. Prevalence of exposure to risk factors was obtained from nationally representative population surveys. Cancer incidence data for 2015 were sourced from national data releases and, where needed, personal communications. PAF calculations were stratified by age, sex and risk factor exposure level and then combined to create summary PAFs by cancer type, sex and country.

**Results:**

Nearly four in ten (37.7%) cancer cases in 2015 in the UK were attributable to known risk factors. The proportion was around two percentage points higher in UK males (38.6%) than in UK females (36.8%). Comparing UK countries, the attributable proportion was highest in Scotland (41.5% for persons) and lowest in England (37.3% for persons). Tobacco smoking contributed by far the largest proportion of attributable cancer cases, followed by overweight/obesity, accounting for 15.1% and 6.3%, respectively, of all cases in the UK in 2015. For 10 cancer types, including two of the five most common cancer types in the UK (lung cancer and melanoma skin cancer), more than 70% of UK cancer cases were attributable to known risk factors.

**Conclusion:**

Tobacco and overweight/obesity remain the top contributors of attributable cancer cases. Tobacco smoking has the highest PAF because it greatly increases cancer risk and has a large number of cancer types associated with it. Overweight/obesity has the second-highest PAF because it affects a high proportion of the UK population and is also linked with many cancer types. Public health policy may seek to mitigate the level of harm associated with exposure or reduce exposure levels—both approaches may effectively impact cancer incidence. Differences in PAFs between countries and sexes are primarily due to varying prevalence of exposure to risk factors and varying proportions of specific cancer types. This variation in turn is affected by socio-demographic differences which drive differences in exposure to theoretically avoidable ‘lifestyle’ factors. PAFs at UK country level have not been available previously and they should be used by policymakers in devolved nations. PAFs are estimates based on the best available data, limitations in those data would generally bias toward underestimation of PAFs. Regular collection of risk factor exposure prevalence data which corresponds with epidemiological evidence is vital for analyses like this and should remain a priority for the UK Government and devolved Administrations.

## Introduction

Over the last decade, age-standardised incidence rates for all cancers combined (International Classification of Diseases version 10 [ICD-10]^1^ C00-C97 excluding C44) have increased by 7% in the UK, with a larger increase in females (8%) than in males (3%).^2,3^ Over the next two decades, incidence rates for all cancers combined are projected to rise by 2% in the UK; this slower pace of increase is in part due to falling smoking rates since the 1970s, the impact of which will be seen most clearly in future decades.^4^ Changes in exposure to risk factors are key drivers of changes in cancer incidence, with improvements in cancer diagnosis and data capture contributing to a lesser extent. Quantifying the contribution of these risk factors indicates the reduction in cancer incidence, which could be achieved through risk exposure reduction or removal.

Efforts to reduce exposure to theoretically modifiable cancer risk factors at individual and societal level may be hampered by the breadth of factors implicated (and possibly limited awareness of some of those factors),^2,3^ and a lack of clarity on which factors have the most impact on cancer risk, and therefore which to prioritise. Risk factors which contribute the most cases to the overall cancer burden are either those with the highest relative risks associated with exposure, those with the highest exposure prevalence in the population, those with the largest number of associated common cancer types, or combinations thereof. Parkin et al.^5^ published novel data on the burden of theoretically avoidable cancer in the UK in 2010. These have informed tobacco, alcohol and obesity policy in the UK, as well as inspiring similar work internationally.^6^

International versions of Parkin^5,7^ work demonstrate how nations differ markedly in the population attributable fractions (PAFs) for specific cancer types and risk factors, and for all cancers and risk factors combined. Several factors underpin true variation between countries. Prevalence of exposure to risk factors varies both with time period and geography. Age and sex profile of cancer cases may vary, often due to different availability of and eligible ages for screening programmes. Morphology breakdowns of individual cancer types (e.g. oesophageal squamous cell carcinoma and adenocarcinoma) vary with risk factor prevalence. Proportions of individual cancer types contributing to the total number of cancers vary due to screening availability and risk factor prevalence. Methodological differences also contribute to PAF differences, for example the relative risks used, calculation methods, and choice of risk factors included.

It is not ideal therefore to use whole-UK PAFs to describe the burden in individual UK countries when many of these factors, most importantly the prevalence of risk factor exposure, is known to vary between them.^8^ It is also important to regularly update widely used figures such as these, to incorporate changes over time in risk factor exposure prevalence, new high-quality evidence on relative risks, changes in the demography of cancer patients, and changes in official classifications of risk factor evidence strength (by the International Agency for Research on Cancer [IARC] and World Cancer Research Fund [WCRF]).^9,10^

This update builds on the methodology devised by Parkin et al.^5^ to provide 2015 PAFs by cancer type and risk factor for the UK overall and for each constituent country. Differences in methodology compared with Parkin et al.^5^ mainly reflect updates to evidence and classifications, and availability and quality of UK country-level exposure prevalence data.

## Materials and methods

### Risk factors included

Combinations of risk factor and cancer type were included in the analysis if they were, at the time of the literature search for the analysis (April 2017), classified by IARC or WCRF as having ‘sufficient’ (IARC) or ‘convincing’ (WCRF) evidence of a causal association.^9–11^ If both IARC and WCRF had issued a classification on a combination of risk factor and cancer type, then the most recently issued classification was used; the source of each classification used is shown in Supplementary Material A. Cancer types with no risk factors classified as having ‘sufficient’ or ‘convincing’ evidence of a causal association (e.g. prostate and testicular cancers) were not included in any PAF calculations, but were included in the all cancers combined total. For oral contraceptives, which increase risk for some cancer types but decrease risk for others, PAFs were calculated only for the cancer types where risk is increased, as the aim of this study is to quantify cancers caused, not the net effect. Ethics approval was not required and the study was performed in accordance with the Declaration of Helsinki.

### PAF formula

For most risk factors, PAFs were calculated using the standard formula described by Parkin et al.^7^

$$\frac{{\left( {{{p}}_1 \times {\mathrm{ERR}}_1} \right) + \left( {{{p}}_2 \times {\mathrm{ERR}}_2} \right) + \left( {{{p}}_3 \times {\mathrm{ERR}}_3} \right) \ldots + \left( {{{p}}_{{n}} \times {\mathrm{ERR}}_{{n}}} \right)}}{{1 + \left[ {\left( {{{p}}_1 \times {\mathrm{ERR}}_1} \right) + \left( {{{p}}_2 \times {\mathrm{ERR}}_2} \right) + \left( {{{p}}_3 \times {\mathrm{ERR}}_3} \right) \ldots + \left( {{{p}}_{{n}} \times {\mathrm{ERR}}_{{n}}} \right)} \right]}}$$

where *p*_1_ is the proportion of the population in exposure level 1 (and so on) and ERR_1_ is the excess relative risk (relative risk – 1) at exposure level 1 (and so on).

Where relative risk (RR) was provided for the presence of/increase in a risk factor when the PAF was to be calculated for the absence of/decrease in that risk factor, ERR was calculated as the natural logarithm of the reciprocal of the RR (ln(1/RR)). Where RR was provided for multiple units when the calculation required ERR per unit, ERR for *x* units was divided by *x* to obtain ERR per unit.

For some risk factors PAFs were obtained from other published studies, as was the case in Parkin et al.^5^ This applied to PAFs for Epstein–Barr virus,^12^ human papillomavirus (HPV),^13,14^ Kaposi sarcoma herpesvirus/human herpesvirus 8 (KSHV/HHV8),^15^ and diagnostic radiation.^16^

Where IARC/WCRF classifications were specific to cancer type subsites, morphological types, or patient age groups (Supplementary Material C), the number of attributable cases was calculated using only those specific attributes, and the PAF used those specific cases as the numerator and the total cases of that overall cancer type as the denominator. For example, the overweight/obesity PAF for stomach cancer uses the attributable cases of gastric cardia stomach cancer, within the total cases of stomach cancer overall. This applied to meningioma and postmenopausal breast cancer for overweight/obesity (denominators were brain tumours and breast cancers); non-cardia stomach cancer and mucosa-associated lymphoid tissue lymphoma for *Helicobacter pylori* (denominators were stomach cancer and non-Hodgkin lymphoma); conjunctiva for HIV (denominator was eye cancer); salivary gland and all leukaemias excluding chronic lymphocytic leukaemia for ionising radiation (denominators were oral cavity cancer and all leukaemias combined); and mucinous ovarian cancer and acute myeloid leukaemia for tobacco (denominators were ovarian cancer and all leukaemias combined).

### Relative risks

RRs were identified through systematic PubMed searches (search terms are shown in Supplementary Material B, selected relative risks and sources are shown in Supplementary Material C). Meta-analyses were the preferred source of RRs, followed by pooled analyses and cohort studies, with case–control studies selected only when no other sources could be found. Within meta- and pooled analyses where multiple analyses were reported, or where more than one meta- or pooled analysis was available, RRs were selected based on characteristics most relevant to the evidence. For example, where statistically significant variation between pooled estimates for different world regions was observed, the Europe/UK estimate was preferred; where there was statistically significant male versus female variation, sex-specific RRs were used; and where confounding was a particular concern, RRs with the most comprehensive adjustment for confounders were selected. Sample size and compatibility with the format of exposure prevalence data were also considered in these decisions, for example, tobacco exposure prevalence was usually defined as cigarette smoking rather than use of other tobacco products, so RRs for cigarettes rather than all tobacco products were used where available. The relative risk of leukaemia associated with ionising radiation exposure was calculated using the formula presented by Parkin et al.^5^

### Risk factor exposure prevalence

For the majority of risk factors analysed, cancer risk increases with higher exposure, the optimum exposure level is nil, and the reference category in the RR sources is ‘unexposed’ (Supplementary Material D). For fibre, physical activity and breastfeeding, increased cancer risk is associated with lower exposure. Fibre exposure prevalence was calculated as deficit against UK Government recommended levels at the time the PAFs were calculated (30 g per day of fibre).^17^ Physical activity exposure prevalence was calculated as deficit against the reference category in the RR source (600 metabolic equivalent [MET]-minutes, or 150 min of moderate-intensity activity per week), because the latest evidence indicates that significant reductions in bowel cancer risk are only achieved at higher physical activity levels than the UK Government recommends.^18^ Breastfeeding exposure prevalence was calculated as absence of the behaviour. While the World Health Organization recommendation (based on benefits to the child) is to breastfeed for 6 months,^19^ the prevalence data available are insufficient to accurately gauge duration of breastfeeding across all the UK countries. For factors where UK Government recommendations are maximum rather than minimum intake (alcohol and processed meat),^20,21^ the optimum exposure was defined as nil.

Prevalence of exposure to risk factors was generally obtained from nationally representative population surveys (Supplementary Material D), at as granular a breakdown of age and sex as the data allowed. Where UK- or Great Britain-wide surveys with a country breakdown provided an adequate sample size for each constituent nation, these were used to afford direct comparability between countries; however, in most cases a separate survey (e.g. national health surveys, which are powered for devolved nations’ analysis) was used for each country. Data were obtained for 2005 for each country wherever possible, providing a ten-year lag between risk exposure and cancer incidence. In some cases it was not possible to match years across countries. Conversions or imputations were made where exposure prevalence data were not available for all cohorts required. These calculations are described in Supplementary Material E; where no calculations are described the data were lifted directly from source with no conversion or imputation required. References are provided in Supplementary Material.

### Incidence

Cancer incidence data for 2015 were obtained for each of the UK constituent countries mainly from their routine annual publications.^22–25^ Generally these publications provided data at the ICD-10 3-digit level. A small number of calculations required incidence data not routinely published: by 4-digit ICD-10 code (e.g. brain, other central nervous system and intracranial tumours), by morphology (e.g. oesophageal squamous cell carcinoma and adenocarcinoma), or for rarer cancer types (e.g. gallbladder and sinonasal cancers). For these calculations, the UK countries’ cancer registries kindly provided appropriate data (Information Services Division Scotland, September 2016, Scotland 2012–2014 incidence data for oesophageal adenocarcinoma, oesophageal squamous cell carcinoma, and mucinous ovarian carcinoma, personal communication; Northern Ireland Cancer Registry, November 2016, Northern Ireland 2010–14 incidence data for oesophageal adenocarcinoma, oesophageal squamous cell carcinoma, and mucinous ovarian carcinoma, personal communication; Office for National Statistics, September 2016, England 2014 incidence data for oesophageal adenocarcinoma, oesophageal squamous cell carcinoma, and mucinous ovarian carcinoma, personal communication; Welsh Cancer Intelligence and Surveillance Unit, June 2016, Wales 2012-2014 incidence data for oesophageal adenocarcinoma, oesophageal squamous cell carcinoma, and mucinous ovarian carcinoma, personal communication).

### Combining PAFs

PAFs for all risk factors combined, for each cancer type and for all cancers combined, were obtained by first applying the first relevant PAF in the sequence shown in Table 1 to the total number of observed cases, to obtain the number of cases attributable to that factor only. The order of risk factors within the sequence does not affect the result of the sum, the order in Table 1 runs from highest to lowest UK PAF within males, females and persons separately. Each subsequent PAF in the sequence was applied only to the number of observed cases not yet explained by the risk factors earlier in the sequence, as described by Parkin et al.^26^ Though the RRs used in the PAFs calculations are generally adjusted and therefore should represent only the effect of the specific risk factor in isolation, residual confounding remains possible. This aggregation method avoids overestimating PAFs for all risk factors combined but does not account for cases caused by exposure to risk factors in combination, e.g. the synergistic effect of tobacco and alcohol on oesophageal cancer risk, or of HPV and tobacco smoking on cervical cancer risk.

**Table 1:** Summary population attributable fractions and attributable cases, by risk factor, sex and country, 2015

	All cancers excluding non-melanoma skin cancer (ICD-10 C00-C97 excluding C44), 2015									
	England		Scotland		Wales		Northern Ireland		UK	
	PAF (%)	Attrib. cases	PAF (%)	Attrib. cases	PAF (%)	Attrib. cases	PAF (%)	Attrib. cases	PAF (%)	Attrib. cases
**Males**										
*Cancer incidence*	152,891		15,184		9,837		4,650		182,562	
Tobacco smoking	17.3	26,375	21.1	3,204	18.6	1,832	17.8	830	17.7	32,242
Overweight and obesity	5.2	7,960	6.0	909	4.6	450	5.3	248	5.2	9,567
Occupation	4.9	7,458	5.8	875	5.3	520	5.0	234	5.0	9,087
Radiation—UV	3.9	5,899	3.9	587	3.7	364	3.8	174	3.8	7,025
Insufficient fibre	3.1	4,713	3.5	529	3.3	322	3.7	171	3.1	5,735
Alcohol	3.0	4,634	3.8	572	3.2	319	3.5	164	3.1	5,689
Infections	3.0	4,539	4.0	608	2.8	279	3.8	178	3.1	5,605
Processed meat	2.0	3,096	2.3	346	2.1	204	2.4	112	2.1	3,758
Radiation—ionising	1.7	2,675	1.6	239	2.0	196	1.7	80	1.7	3,190
Air pollution	1.1	1,636	1.0	146	0.8	82	0.8	38	1.0	1,901
Insufficient physical activity	0.5	794	0.6	85	0.5	51	0.6	27	0.5	957
All of the above	38.0	58,141	43.3	6,567	39.0	3,838	39.9	1,856	38.6	70,425
**Females**										
*Cancer incidence*	146,862		16,266		9,251		4,606		176,985	
Tobacco smoking	12.1	17,738	15.6	2,532	13.4	1,241	11.3	519	12.4	22,029
Overweight and obesity	7.5	11,036	7.6	1,244	6.4	590	7.0	324	7.5	13,194
Infections	4.0	6,083	5.1	832	3.9	364	4.4	202	4.2	7,481
Radiation—UV	3.8	5,541	3.5	570	3.4	311	3.4	157	3.7	6,579
Alcohol	3.5	5,202	3.3	538	3.3	301	3.5	163	3.5	6,205
Insufficient fibre	3.3	4,917	3.5	564	3.4	316	3.5	161	3.4	5,958
Occupation	2.4	3,528	2.8	462	2.6	241	2.5	114	2.5	4,338
Radiation—ionising	2.1	3,128	1.9	314	2.4	221	2.2	100	2.1	3,764
Not breastfeeding	1.4	2,117	1.5	248	1.4	132	1.9	86	1.5	2,582
Air pollution	1.0	1,442	0.9	142	0.8	74	0.7	32	1.0	1,690
Processed meat	0.9	1,330	0.9	145	0.8	73	1.0	46	0.9	1,594
Postmenopausal hormones	0.7	1,089	0.8	132	1.2	107	0.9	43	0.8	1,371
Insufficient physical activity	0.5	801	0.5	86	0.5	49	0.5	25	0.5	959
Oral contraceptives	0.5	667	0.5	79	0.3	32	0.7	30	0.5	807
All of the above	36.4	53,480	39.7	6,455	36.5	3,373	36.1	1,663	36.8	65,130
**Persons**										
*Cancer incidence*	299,753		31,450		19,088		9,256		359,547	
Tobacco smoking	14.7	44,113	18.2	5,736	16.1	3,073	14.6%	1,349	15.1	54,271
Overweight and obesity	6.3	18,996	6.8	2,153	5.4%	1,040	6.2	572	6.3	22,761
Radiation—UV	3.8	11,440	3.7	1,157	3.5	675	3.6	332	3.8	13,604
Occupation	3.7	11,078	4.4	1,373	4.0	765	3.8	353	3.8	13,558
Infections	3.5	10,622	4.6	1,441	3.4	643	4.1	380	3.6	13,086
Alcohol	3.3	9,836	3.5	1,110	3.3	621	3.5	327	3.3	11,894
Insufficient fibre	3.2	9,630	3.5	1,093	3.3	638	3.6	332	3.3	11,693
Radiation—ionising	1.9	5,803	1.8	553	2.2	417	1.9	180	1.9	6,954
Processed meat	1.5	4,426	1.6	490	1.4	276	1.7	159	1.5	5,352
Air pollution	1.0	3,078	0.9	288	0.8	156	0.8	70	1.0	3,591
Not breastfeeding	0.7	2,117	0.8	248	0.7	132	0.9	86	0.7	2,582
Insufficient physical activity	0.5	1,595	0.5	171	0.5	100	0.6	51	0.5	1,917
Postmenopausal hormones	0.4	1,089	0.4	132	0.6	107	0.5	43	0.4	1,371
Oral contraceptives	0.2	667	0.2	79	0.2	32	0.3	30	0.2	807
All of the above	37.3	111,722	41.5	13,038	37.8	7,207	38.0	3,519	37.7	135,507

## Results

Summary results are presented in Tables 1 and 2. More detailed results by risk factor–cancer type combination, cancer type, sex and country are presented in Supplementary Material F.

**Table 2:** Summary population attributable fractions and attributable cases, by cancer type, sex and country, 2015

Cancer type and ICD-10 code		All risk factors combined														
		England			Scotland			Wales			Northern Ireland			UK		
		*M*	*F*	*P*	*M*	*F*	*P*	*M*	*F*	*P*	*M*	*F*	*P*	*M*	*F*	*P*
Oral cavity (C00–C06)	PAF (%)	52.9	34.3	46.3	53.3	32.8	46.2	53.3	32.0	46.4	52.9	32.3	46.8	52.9	34.0	46.3
	Attrib. cases	1,422	515	1,941	188	63	251	44	27	77	22	13	38	1,676	618	2,308
Nasopharynx (C11)	PAF (%)	85.3	84.3	84.9	85.1	84.5	84.9	85.9	85.0	85.8	85.7	84.6	85.5	85.3	84.3	85.0
	Attrib. cases	101	51	153	20	6	25	13	2	15	4	1	5	138	60	198
Pharynx (C09, C10, C12–C14)	PAF (%)	90.2	81.4	88.3	90.5	82.2	88.6	90.5	81.5	88.9	90.4	80.4	88.4	90.2	81.5	88.4
	Attrib. cases	1,621	472	2,100	218	67	286	112	26	139	65	18	84	2,017	584	2,609
Oesophagus (C15)	PAF (%)	60.9	54.4	58.7	61.0	55.2	58.9	59.3	52.3	56.9	55.9	54.9	55.4	60.7	54.4	58.6
	Attrib. cases	3,068	1,306	4,367	363	179	542	188	85	273	72	41	113	3,691	1,612	5,295
Stomach (C16)	PAF (%)	56.4	47.5	53.1	67.6	57.4	64.0	51.3	43.7	48.2	66.0	63.8	65.0	57.3	48.6	54.2
	Attrib. cases	2,009	921	2,925	260	129	390	137	68	203	90	41	130	2,496	1,159	3,649
Bowel (C18-C20)	PAF (%)	57.0	50.8	54.1	59.3	52.3	56.0	56.7	50.1	53.8	58.8	51.5	55.6	57.2	50.9	54.3
	Attrib. cases	10,923	7,895	18,796	1,170	887	2,056	724	492	1,215	375	249	624	13,193	9,523	22,691
Anus (C21)	PAF (%)	88.7	92.5	91.3	88.7	92.5	91.2	88.7	92.5	90.9	88.7	92.5	91.5	88.7	92.5	91.3
	Attrib. cases	357	789	1,146	43	87	130	23	34	57	5	15	20	428	925	1,353
Liver (C22)	PAF (%)	53.0	39.3	48.3	55.7	44.9	52.2	51.7	35.3	47.0	51.0	36.9	45.6	53.2	39.6	48.5
	Attrib. cases	1,579	666	2,255	220	90	311	115	34	150	41	20	61	1,955	811	2,778
Pancreas (C25)	PAF (%)	33.9	28.5	31.2	36.2	31.8	34.0	35.5	29.2	32.2	35.0	27.0	31.3	34.2	28.7	31.5
	Attrib. cases	1,415	1,179	2,596	144	131	276	85	79	164	51	34	86	1,694	1,424	3,120
Gallbladder (C23)	PAF (%)	12.5	23.0	19.9	13.7	24.7	21.8	9.6	18.1	15.5	12.7	21.1	20.7	12.4	22.8	19.9
	Attrib. cases	31	140	171	3	14	17	1	5	7	0	4	4	35	163	198
Larynx (C32)	PAF (%)	73.4	66.0	72.1	74.9	69.8	73.9	76.7	67.3	75.0	74.2	63.0	72.4	73.8	66.5	72.5
	Attrib. cases	1,120	238	1,360	177	40	217	97	20	117	51	9	61	1,444	308	1,754
Lung (C33–C34)	PAF (%)	81.8	74.8	78.8	81.7	76.7	79.4	83.8	77.0	80.8	80.6	68.4	75.4	81.9	75.0	78.9
	Attrib. cases	16,369	13,184	29,631	2,068	1,890	3,969	1,081	907	1,993	541	389	935	20,060	16,372	36,532
Mesothelioma (C45)	PAF (%)	97.0	82.5	94.4	97.0	82.5	94.4	97.0	82.5	94.4	97.0	82.5	94.4	97.0	82.5	94.4
	Attrib. cases	1,895	321	2,212	180	18	196	110	9	117	35	5	40	2,220	353	2,565
Melanoma (C43)	PAF (%)	88.6	84.8	86.8	83.0	83.0	84.9	91.0	81.0	86.1	83.5	81.4	82.5	88.5	84.4	86.5
	Attrib. cases	5,899	5,541	11,440	561	570	1157	364	311	675	174	157	332	7025	6579	13,604
Kaposi sarcoma (C46.1)	PAF (%)	100.0	100.0	100.0	100.0	0.0	100.0	100.0	100.0	100.0	100.0	100.0	100.0	100.0	100.0	100.0
	Attrib. cases	125	17	142	7	0	7	2	1	3	3	1	4	137	19	156
Breast (C50)	PAF (%)	0.0	23.0	22.9	0.1	24.0	24.0	0.1	23.0	22.9	0.0	24.5	24.4	0.0	23.1	23.0
	Attrib. cases	—	10,523	10,523	—	1,139	1,139	—	641	641	—	357	357	—	12,659	12,659
Vulva (C51)	PAF (%)	0.0	68.8	68.8	0.0	68.8	68.8	0.0	68.8	68.8	0.0	68.8	68.8	0.0	68.8	68.8
	Attrib. cases	—	744	744	—	97	97	—	55	55	—	25	25	—	921	921
Vagina (C52)	PAF (%)	0.0	75.0	75.0	0.0	75.0	75.0	0.0	75.0	75.0	0.0	75.0	75.0	0.0	75.0	75.0
	Attrib. cases	—	148	148	—	16	16	—	5	5	—	6	6	—	174	174
Cervix (C53)	PAF (%)	0.0	99.8	99.8	0.0	99.8	99.8	0.0	99.8	99.8	0.0	99.8	99.8	0.0	99.8	99.8
	Attrib. cases	—	2511	2511	—	378	378	—	149	149	—	81	81	—	3119	3119
Uterus (C54–C55)	PAF (%)	0.0	34.4	34.4	0.0	34.9	34.9	0.0	30.1	30.1	0.0	28.4	28.4	0.0	34.0	34.0
	Attrib. cases	—	2561	2561	—	279	279	—	145	145	—	70	70	—	3056	3056
Ovary (C56)	PAF (%)	0.0	11.1	11.1	0.0	12.1	12.1	0.0	11.2	11.2	0.0	12.6	12.6	0.0	11.2	11.2
	Attrib. cases	—	641	641	—	69	69	—	40	40	—	17	17	—	766	766
Penis (C60)	PAF (%)	63.3	0.0	63.3	63.3	0.0	63.3	63.3	0.0	63.3	63.3	0.0	63.3	63.3	0.0	63.3
	Attrib. cases	329	—	329	45	—	45	18	—	18	13	—	13	404	—	404
Bladder (C67)	PAF (%)	50.6	43.2	48.6	51.7	47.1	50.4	54.0	46.2	51.9	48.7	37.1	45.2	50.8	43.6	48.9
	Attrib. cases	3,112	1,007	4,125	285	131	417	241	84	326	70	26	96	3,708	1,247	4,964
Kidney (C64-C66, C68)	PAF (%)	32.1	36.1	33.5	32.9	38.1	34.8	30.7	33.1	31.5	31.7	33.5	32.2	32.1	36.1	33.5
	Attrib. cases	2,068	1,404	3,467	231	154	385	111	68	179	72	40	112	2,483	1,666	4,142
Thyroid (C73)	PAF (%)	9.8	8.8	9.1	10.0	9.6	9.7	8.5	7.8	8.1	11.0	8.1	8.9	9.8	8.8	9.1
	Attrib. cases	80	197	278	8	19	27	3	6	9	3	4	7	94	227	321
Myeloma (C90)	PAF (%)	15.8	10.5	13.6	16.8	11.5	14.6	13.8	8.8	11.4	16.3	9.9	13.4	15.8	10.5	13.6
	Attrib. cases	425	205	630	47	22	69	21	12	33	13	6	19	505	246	751
Hodgkin lymphoma (C81)	PAF (%)	40.1	40.7	40.3	41.2	40.1	40.7	43.0	40.0	41.6	41.8	37.4	39.9	40.4	40.5	40.4
	Attrib. cases	414	305	719	30	30	60	27	23	49	15	10	25	486	367	853
Non-Hodgkin lymphoma (C82–C85, C96)	PAF (%)	3.4	3.3	3.4	4.2	4.8	4.5	3.4	3.3	3.4	3.9	3.8	3.8	3.5	3.4	3.5
	Attrib. cases	122	64	185	22	24	46	12	10	22	7	6	12	260	211	472
Leukaemia (C91-C95)	PAF (%)	11.3	12.9	12.0	13.0	15.7	14.0	11.0	12.5	11.5	12.9	13.5	13.2	11.5	13.1	12.1
	Attrib. cases	567	444	1,009	47	38	85	43	29	72	16	14	30	673	525	1195
Brain & other central nervous system (C70–C72)	PAF (%)	0.0	4.8	2.5	0.1	6.3	3.5	0.1	3.5	2.0	0.0	4.9	2.6	0.0	4.8	2.5
	Attrib. cases	2	228	230	0	34	34	0	16	17	0	10	10	3	288	291
All excl non-melanoma skin cancer (C00–C97 excl C44)	PAF (%)	38.0	36.4	37.3	43.3	39.7	41.5	39.0	36.5	37.8	39.9	36.1	38.0	38.6	36.8	37.7
	Attrib. cases	58,141	53,480	111,722	6,567	6,455	13,038	3,838	3,373	7,207	1,856	1,663	3,519	70,425	65,130	135,507

### UK

Nearly four in ten (37.7%) cancer cases in 2015 in the UK were attributable to known risk factors. The proportion was around two percentage points higher in UK males (38.6%) than in UK females (36.8%). Excluding sex-specific cancer types (cervix, ovary, uterus, vagina, vulva, penis [prostate and testicular have no risk factor-attributable cases in these calculations]) and breast cancer, the proportion was much higher in UK males (36.4%) than in UK females (25.6%).

The attributable proportion for all cancers combined was highest in Scotland (41.5% for persons) and lowest in England (37.3% for persons). Between-country variation was marginally larger for males than for females, with around five (males) and four (females) percentage points between highest and lowest.

Tobacco smoking contributed by far the largest proportion of attributable cancer cases, accounting for 15.1% of all cases in the UK in 2015. Smoking had the highest PAF in all the UK countries. The proportion was higher in UK males (17.7%) than in UK females (12.4%), reflecting higher smoking prevalence in males in 2005. The tobacco smoking-attributable proportion of cancer cases was highest in Scotland (18.2% for persons) and lowest in Northern Ireland (14.6% for persons). The cancer types with the highest PAFs for tobacco smoking were lung (72.2% for UK persons) and larynx (64.0% for UK persons).

Overweight and obesity was the second-largest preventable cause of cancer in the UK and accounted for 6.3% of all cases in the UK in 2015. This factor was second-highest in all the UK constituent countries. The proportion was higher in UK females (7.5%) than in UK males (5.2%), and was highest in Scotland (6.8% for persons) and lowest in Wales (5.4% for persons). The cancer types with the highest PAFs for overweight and obesity were uterine for females (34.0% for UK females) and oesophagus for males (31.3% for UK males).

UV radiation and occupational risks contributed the next-highest proportions of attributable cases (both 3.8% in UK persons), both with less than one percentage point difference in PAFs between highest (Scotland for occupation, England for UV) and lowest (England for occupation, Wales for UV) countries.

Exposure to infections, alcohol drinking and insufficient dietary fibre each contributed 2–4% of attributable cancer cases in UK persons. The remaining factors contributed less than 2% each.

For 10 cancer types, including two female-specific sites, more than 70% of cases in UK persons were attributable to known risk factors: Kaposi sarcoma (100%), cervical (99.8%), mesothelioma (94.4%), anal (91.3%), pharyngeal (88.4%), nasopharyngeal (85.0%), melanoma (86.5%), lung (78.9%), vaginal (75.0%) and laryngeal (72.5%).

### England

Almost four in ten (37.3%) cancer cases in 2015 in England were attributable to known risk factors. The proportion differed only marginally between England males (38.0%) and females (36.4%), in contrast to the other UK countries where the sex difference was larger. The overall PAF was lowest in England males and second-lowest in England females, when comparing between countries.

Tobacco smoking contributed the largest proportion of England’s attributable cancer cases (14.7%). This was the second-lowest tobacco smoking-attributable proportion among the UK countries. Overweight and obesity contributed the second-highest proportion of cases in England (6.3%) and this proportion was second-highest among the UK countries.

England had the joint-largest (with Scotland) sex difference in the UK in alcohol PAFs, with a lower PAF for males (3.0%) than for females (3.5%).

Among the UK countries, England had the highest PAFs for UV radiation and air pollution. England had the lowest or joint-lowest PAF among the UK countries for a number of risk factors, including alcohol drinking, insufficient dietary fibre and occupational risks.

These PAF differences reflect risk factor exposure prevalence, for example England’s current smoking prevalence was the lowest in the UK in 2005, and its overweight and obesity prevalence was the highest in the UK in 2005.

### Scotland

Around four in ten (41.5%) cancer cases in 2015 in Scotland were attributable to known risk factors. The proportion was nearly four percentage points higher in Scotland males (43.3%) than in females (39.7%). The overall PAF was the highest among the UK countries for both males and females.

These PAF differences reflect risk factor exposure prevalence, but also proportion of specific cancer types in the all cancers combined total. For example, 2005 smoking prevalence is not markedly higher in Scotland than in the other UK countries, but Scotland has a higher proportion of lung cancer cases in its all cancer combined total.

Tobacco smoking contributed the largest proportion of attributable cancer cases in Scotland (18.2%). This was by far the largest tobacco smoking-attributable proportion among the UK countries, around two percentage points higher than the next-highest country. Overweight and obesity contributed the second-highest proportion of cases (6.8%) and again this proportion was highest among the UK countries, though the between-country variation here was smaller.

Scotland had the joint-largest (with England) sex difference in the UK in alcohol PAFs, but in the opposite direction to England with a higher PAF in males (3.8%) than in females (3.3%).

Scotland had the lowest PAF in the UK for only one risk factor: ionising radiation. For all other risk factors Scotland had the highest or second-highest PAF in the UK.

### Wales

Nearly four in ten (37.8%) cancer cases in 2015 in Wales were attributable to known risk factors. The proportion was more than two percentage points higher in Wales males (39.0%) than in females (36.5%). The overall females PAF was second-highest among the UK countries.

Tobacco smoking and overweight and obesity contributed the highest proportions of cases (16.1% and 5.4% respectively). The overweight and obesity PAF was lowest in Wales compared with the other UK countries.

Wales had the highest PAFs in the UK for ionising radiation and postmenopausal hormones, and the lowest or joint-lowest PAFs in the UK for processed meat, infections, UV radiation, alcohol, physical activity, air pollution and not breastfeeding.

Risk factor exposure prevalence again underpins these results: Wales had particularly low obesity prevalence in 2004/2005 (although overweight prevalence was similar to other UK countries), and radon levels are slightly higher in Wales than elsewhere in the UK.

### Northern Ireland

Nearly four in ten (38.0%) cancer cases in 2015 in Northern Ireland were attributable to known risk factors. The proportion was nearly four percentage points higher in Northern Ireland males (39.9%) than in females (36.1%).

Northern Ireland’s tobacco PAF was the lowest among the UK countries for males and females combined, though this was mainly driven by the between-country pattern in females. Northern Ireland had the largest sex difference in the UK in tobacco smoking PAFs, with the PAF around 50% higher in males (17.8%) than in females (11.3%).

Tobacco smoking and overweight and obesity contributed the highest proportions of cases in Northern Ireland (14.6% and 6.2% respectively).

Northern Ireland had the highest or joint-highest PAFs in the UK for processed meat, insufficient dietary fibre, insufficient physical activity, oral contraceptives, not breastfeeding, and alcohol—though for all these factors the difference was very small. It had the joint-lowest (with Wales) PAF in the UK for air pollution.

Prevalence of diet and physical activity risk factors was higher in Northern Ireland in 2005 compared with the other UK countries. Although Northern Ireland’s air pollution concentrations were second-lowest to Scotland, the higher proportion of lung cancer in Scotland meant more air pollution-attributable cases there.

## Discussion

### Variation by sex and country

Variation by UK country and sex was generally only a few percentage points, so these differences should be interpreted cautiously.

Males have higher prevalence of exposure to risk factors than do females, across almost all risk factors and UK countries. Tobacco smoking, overweight and obesity, meat-eating, and alcohol drinking are more common and/or at higher levels in men than in women^27–30^). Fibre is a notable exception, with lower intake, and accordingly higher PAFs, in females than in males. The male excess in risk factor exposure is generally not offset by the female-only cancer types, with the exceptions of overweight and obesity, infections, and ionising radiation, where PAFs are higher in females than in males mainly because of sex-specific cancers, some of which have high PAFs. Nor is men’s higher risk factor exposure offset by female-only risk factors such as exogenous hormone use and non-breastfeeding.

The relative risk of cancer associated with risk factor exposure is often higher in males than in females (although this may relate to sample size and statistical power, discussed in more detail below), so even where exposure levels are similar, the estimated population impact is higher in males than in females. The all cancers combined total in males comprises a higher proportion of tobacco smoking-associated cancer types with high individual PAFs, while in females some of the largest contributors to the all cancers combined total have reasonably low individual PAFs (e.g. 23.0% for breast cancer). Sex-specific cancers contribute much more to the total PAF for females than for males, with breast cancer accounting for most of the difference.

Differences between countries in the all cancers combined PAFs are due to a combination of two related factors: risk factor exposure prevalence and proportions of specific cancer types. Differences in risk factor exposure prevalence between countries are to some extent a reflection of data availability and quality in each nation, and comparisons between countries’ PAFs should be made with this in mind. Any true differences probably reflect demographic differences which drive those ‘lifestyle’ differences. For example, areas with higher levels of socioeconomic deprivation have higher tobacco smoking rates^31^; areas with larger populations which eschew alcohol for faith reasons have lower alcohol-drinking rates.^32^ Further, many lifestyle ‘choices’ are driven by environmental/societal factors such as food pricing and availability, and susceptibility to these factors varies with socioeconomic position.^33^ Differences in risk factor exposure prevalence between countries are generally not large, with the exception of *H. pylori* which may reflect both differing deprivation levels across the UK and artefact due to differing data periods.^34^ Factors beyond individual-level control also vary between countries, for example, the predominant occupation groups and air pollution levels. Geographical variation, for example, in radon and UV exposure levels is not controllable (although individuals and Government can take steps to ameliorate the risk associated with those factors). Similarly, having a higher proportion of workers in ‘cancer risk’ industries may not translate to a higher proportion of occupation-related cancers, as employers and Government can implement risk-reduction policies; however, the country-specific occupation PAFs presented here account only for variation in workforce size, not for possible variation in workplace safety.

### Variation by risk factor

Risk factors with the largest PAFs are either those with the highest relative risks associated with exposure, those with the highest exposure prevalence in the population, those with the largest number of associated common cancer types, or combinations thereof. For example, tobacco smoking rates are lower than alcohol drinking rates but tobacco smoking has a much greater impact on cancer risk, and a much larger number of cancer types associated with it, leading to a much higher PAF.

### Comparison with other relevant studies

The results reported here are overall in line with those from similar studies, though methodological differences—different groups of risk factors used, different time periods and different relative risk sources—preclude direct comparisons. For all modifiable risk factors and all cancers combined where the UK 2015 PAF was 37.7%, other reported PAFs include 42.0% in the US in 2014;^35^ 40.8% in Alberta, Canada in 2012;^36^ and 31.9% in Australia in 2010.^37^ In all these studies the preventable proportion was higher in males than in females, with the gap widest in Canada (3.7 percentage points) and smallest in the US (1.0 percentage points), in line with the 1.8 percentage point sex difference reported here. Tobacco contributed the highest proportion of preventable cases across the board (PAFs ranging from 19.0% in the US 2014 to 13.4% in Australia 2010; 15.1% in the UK 2015), with between-country variation reflecting method differences and, arguably, temporal changes in smoking prevalence worldwide. Overweight and obesity was the second-biggest cause of cancer after tobacco in the US 2014 (PAF 7.8%) and UK 2015 (PAF 6.3%), and ranked third in Canada 2012 (PAF 4.3%) and fourth in Australia 2010 (PAF 3.4%). Between-country variation here mainly reflects geographical and temporal differences in overweight/obesity prevalence, and is in line with Arnold and colleagues’ global overweight/obesity PAFs calculations for 2012,^38^ reinforcing their conclusion that the UK has among the highest proportion of overweight/obesity-associated cancers in the world.

The obvious reference point for this work is the UK PAFs published by Parkin and colleagues in 2011.^5^ The all cancers combined PAF for UK persons presented here (37.7%) is almost five percentage points lower than the equivalent figure obtained by Parkin et al. (42.7%).^26^ This does not represent a direct temporal change: changes in risk factor prevalence, cancer incidence and study methodology have all contributed to this difference.

This study has built on Parkin et al.^5^ Risk factors with probable/limited evidence for associations with specific cancer types were included by Parkin et al.,^5^ but have not been included in this study. The difference in inclusion criteria for risk factor-cancer type combinations partly explains the lower PAFs seen here compared with Parkin et al.'s^5^ work, though this effect is reduced by the addition of new combinations which have been classified as sufficient/convincing over the last 6 years.

This study has used specific cancer type subsites, morphological types and patient age groups where evidence of causality was specific to those attributes, where Parkin et al.^5^ often used entire cancer types in their calculations.

The evidence base on relative cancer risk for specific risk factor-cancer type associations has improved since Parkin et al.'s^5^ study, with many more meta-analyses available now. These gold standard evidence syntheses have been used in preference to single studies wherever possible in this work. The meta-analyses used in this study typically report lower relative risks than the single studies used by Parkin et al.,^5^ and this is an important explanation for the difference in PAFs between the studies.

Risk factor exposure prevalence is different in this study compared with Parkin et al.,^5^ and this explains a large part of the difference in PAFs obtained. Risk factor exposure prevalence changed between 2000 (ref. ^5^) and 2005. In this period shifts both towards optimal population prevalence (e.g. reduction in smoking prevalence) and away from it (e.g. increase in overweight and obesity prevalence). This study used risk factor exposure prevalence data from each UK constituent country where available, where Parkin et al.^5^ typically used England or Great Britain as a proxy for the whole UK.

In Parkin et al.'s^5^ estimated 2010 cancer incidence data, smoking-related cancers contributed 52% of the males all cancers combined total, and 43% of the females all cancers combined total. In this study’s observed 2015 cancer incidence data, these proportions were 50% and 42%. Therefore even if the 2015 cancer type PAFs were identical to the 2010 cancer type PAFs, the all cancers combined PAF would be lower simply because the proportion of smoking-related cancers in the all cancers combined denominator is lower.

The largest methodological difference between Parkin et al.^5^ and the current work is in tobacco smoking—but method differences apart, tobacco smoking PAFs have fallen over this time because of reductions in smoking prevalence. Calculating the 2010 tobacco PAF using 2000 smoking prevalence with the same method as in this study produces PAFs of 19.9%, 12.2% and 16.1% for UK males, females and persons respectively—markedly higher than the corresponding 2015 PAFs of 17.7%, 12.4% and 15.1%.

Differences in methodology do also contribute to the different PAFs between studies. Here, lower RRs from meta-analyses have been used, while Parkin et al.^5^ used higher RRs mainly from single studies, and this is a key driver of the PAF differences. The use of survey-reported smoking prevalence rather than notional smoking prevalence as used by Parkin et al.^5^ made a smaller difference. The main benefit of using notional prevalence is that latency between smoking and cancer does not need to be defined,^39,40^ and the choice of latency in the current work is almost certainly too short for tobacco smoking. To use a different lag for smoking than for the other risk factors would not have been systematic, given the similarly sparse data on latency for smoking and for the other risk factors included in this work. Aside from this latency-related benefit, using notional in incidence PAF calculations across multiple cancer types is problematic because it represents a substantial deviation from the original purpose of the method and therefore requires many assumptions which were not considered reliable enough for use in the current study.

There are other methodological differences between this study and Parkin et al.^5^ which influenced the PAFs, but these are relatively small. The UV radiation PAF was calculated using several theoretically UV-unexposed/less UV-exposed groups rather than the single less-exposed birth cohort used in the original project, in an effort to reduce the impact of overdiagnosis in skin cancer which is thought to have increased over time.^41^ However these PAFs probably reflect increased diagnosis as well as true increased incidence, and the relative contribution of each is impossible to assess. Alcohol consumption and breastfeeding prevalence were calculated as categorical rather than continuous variables, in order to minimise the amount of manipulation and assumption around the exposure prevalence data and to better match the sources of relative risks. Moderate physical activity was defined as 4 METs rather than 6 METs as used by Parkin et al.,^5^ again to better match the source of relative risks and reflect the World Health Organization definition of moderate physical activity.^42^ The optimum level of physical activity was defined as exceeding, rather than just reaching, 10 MET-hours per week, because recent evidence suggests bowel cancer risk is only reduced at substantially higher levels.^18^ Meat pies and pastries and other meat and meat products were included with processed meat in this study where they were excluded from meat calculations in Parkin et al.,^5^ because the definition of these categories places them fairly clearly in the processed category and they make up a sizeable proportion of processed meat intake. The optimum fibre intake was defined as 30 g/day rather than 23 g/day to reflect the current guidelines which form the context for policymakers’ use of the current study’s results. Cases caused by oral contraceptive use were included in the all cancers combined PAF where oral contraceptives were excluded altogether from the all cancers combined PAF in ref. ^5^ because of the net protective effect of oral contraceptive use. A net protective effect was observed in the current study (around 4,400 cases prevented and around 800 cases caused), but there is a burden of preventable cases nonetheless and the aim of this work was to quantify preventable cases. The effect of including the causal effect of oral contraceptives in the overall PAF is minimal: omitting oral contraceptives entirely from the UK persons all cancers combined PAF would reduce that PAF by only 0.2 percentage points.

### Strengths and limitations

This work provides UK and constituent country-level PAF estimates for the full compendium of risk factors where evidence of a causal role in cancer development is sufficient/convincing. PAFs at this level have not been available previously and they will be useful for policymakers in devolved nations. Further, at a UK level this work updates the original evidence from Parkin et al.,^5^ and this update is timely given changes in risk factor exposure prevalence and developments in epidemiological evidence.

The PAFs presented here are estimates based on the best available data; therefore, the PAFs should be interpreted with the limitations of the source data (and the limitations of the calculations made on those data) in mind. Most of these limitations would bias toward underestimation of PAFs in the current work. Traditional confidence intervals cannot be provided due to the multiple components in the PAF calculation. Sensitivity analyses—using the upper and lower confidence intervals of the RR and risk factor exposure prevalence data to calculate the highest- and lowest-possible PAFs—were conducted for most risk factor-cancer type combinations, as colleagues using the same PAF calculation method have done.^5,6^ However, as in these colleagues’ work, the results of those analyses are not reported here lest they be misleading, the ranges implying precision though they do not take into account all the possible biases operating on the components of the PAF calculations.^43^

Restricting to risk factors with IARC/WCRF-classified sufficient/convincing evidence of a causal link with cancer is likely to underestimate the true PAF, as genuine risk factor-cancer type combinations may not yet be clear. For example, evidence is mounting for a causal association between obesity and risk of advanced prostate cancer,^44,45^ and were this risk factor–cancer type combination to be included in the present calculations, the overall PAF for males would increase slightly. For some risk factor–site–sex combinations, the association is not statistically significant in the latest evidence, so the RR has been set to 1 in the PAF calculations (Supplementary Material C) resulting in no attributable cases. This may in some cases reflect lack of statistical power (particularly for rarer cancer types and less prevalent behaviours) rather than a genuine lack of association. Excluding all non-significant RRs has almost certainly resulted in conservative PAFs.

Comparison between risk factors is only as reliable as the relative risk evidence available, and in most cases confounding cannot be completely ruled out. For example, the alcohol PAFs are likely to be underestimates as ‘unexposed’ reference groups in this literature often include ex- and occasional drinkers, which dilutes the observed effect of alcohol drinking on cancer risk.^46^ The relative risk figures used were identified and selected systematically but different choices here would influence the PAFs.

Perhaps one of the most vexed issues in PAF calculation is latency, and the results presented here are certainly affected by using a blanket ten-year latency period across all risk factors. PAF calculations are limited by the availability of relative risk and exposure prevalence data for the relevant period. There would be bias in calculating a PAF assuming 30-year latency, with poor exposure prevalence data and relative risk from a study with only a ten-year follow-up, just as there is in calculating a PAF using good exposure prevalence and appropriate relative risk data assuming a 10-year latency which is too short. Clear data on latency between exposure and cancer development are lacking, moreso for some cancer types than others, and using bespoke lags for each cancer type in this study would have been unsystematic and reduced comparability between risk factors. Tobacco smoking has the most evidence for a longer latency and as tobacco smoking prevalence is falling, the tobacco smoking PAF is almost certainly an underestimate. Despite this, calculating the UK 2015 PAF for tobacco smoking using a 20-year latency produces only a 1 percentage point increase compared with the 10-year latency 2015 PAF, therefore supporting the use of a shorter latency with higher quality data for the UK countries.

PAFs for individual risk factor–cancer type combinations represent the fraction of that cancer attributable to that risk factor in isolation, when the effect of other risk factors has been controlled for in the relative risk figure. Control for confounding is easier for some risk factors and cancer types than others. The method of summing individual PAFs to reach the all factors combined total for each risk factor avoids overestimation by applying PAFs sequentially only to the cases not attributed for by factors earlier in the sequence. The issue of cancer cases with more than one cause is distinct from that of cancer cases caused by the synergistic effects of risk factors in combination. For example, the effect of tobacco smoking and alcohol drinking together on oesophageal cancer risk,^47^ or on radon and smoking together on lung cancer risk,^48^ is several times greater than the effects of these factors individually. Synergistic effects have not been included in the calculations reported here for several reasons. National survey data on prevalence of combined risk factor exposure are not sufficiently detailed for use in PAF calculations, and IARC and WCRF do not comment explicitly on synergistic effects so those cancer type-risk factor combinations cannot be evaluated against our inclusion criteria. Further, there is a strong possibility of double-counting if synergistic effects are included: residual confounding in the RRs for individual risk factors (a particular concern for alcohol RRs being confounded by tobacco) would mean that some of the ‘alcohol-only’ cases actually do reflect alcohol and tobacco in combination; adding ‘official’ alcohol and tobacco synergy cases to this would arguably risk overestimating the PAF.

Risk factor exposure prevalence data from surveys is prone to self-reporting errors, particularly underestimation of exposure.^49^ Throughout the analysis some datapoints for specific countries or time periods were not available, and so imputation, estimation and extrapolation (see Supplementary Material E) were required to fill those gaps. This has particularly affected the devolved nations’ results, and this project demonstrates the value of collecting risk factor exposure prevalence data consistently across countries, regularly, and in a format which facilitates linkage with epidemiological data in order to calculate the most accurate PAFs.

Operationalising overweight and obesity prevalence, alcohol consumption, and breastfeeding prevalence as categorical rather than continuous variables is likely to have overestimated the PAFs for these risk factors. However, available exposure prevalence and relative risk data were overwhelmingly categorical, so converting to continuous data would have introduced further uncertainty. RRs comparing categories of people will overestimate the effect for those very near to the category boundary and underestimate the effect for those furthest away from it. If the exposure prevalence distribution is left-skewed (more people near the boundary with optimum exposure), as is the case for overweight and obesity, then the PAF is likely to be an overestimate. This is less of a concern if the within-category distribution is similar in the RR source and the exposure prevalence. However, this information is rarely reported and so the risk of PAF overestimation on this basis cannot be quantified. More accurate PAFs could be calculated if relative risks and exposure prevalence were reported continuously rather than categorically.

The physical activity PAF may be an overestimate as those people achieving 600 + MET-min in less than 5 days were classified as inadequately active. Exposure prevalence data were provided as days when 30 + min moderate physical activity was achieved, rather than total minutes per week. Exposure prevalence data are collected in a format matching the current UK Government physical activity guidelines but the latest evidence shows that these guidelines need to be exceeded quite substantially to impact bowel cancer risk.

Air pollution PAFs are based on exposure prevalence in 2010, although outdoor air pollution levels have decreased markedly over past decades.^50^ However in the absence of firm evidence on latency in this area, erring toward underestimating the PAF was preferred.

The occupation chapter in the original UK attributable cancers project was based on a large separate piece of work and it was beyond the scope of this update to re-create that work. The method used to derive country-level PAFs for occupation here is crude but the results are not unexpected: Among the UK countries Scotland and Wales have the highest overall occupation PAFs and those countries have the highest proportions of the workforce in industries with the highest exposure to cancer risk factors (construction and manufacturing, specifically mining). Removing shiftwork and non-melanoma skin cancer from the PAF estimates in the original attributable cancers project report was offset by adjusting for country-specific occupational exposure levels, so the all cancers combined occupation PAF for the UK has remained similar to the original estimate.

Oral contraceptives calculations use the most recent freely available data with appropriate age breakdowns, from 2010 to 2012. These were assumed accurate for ‘current’ use in 2015 (as the RRs are for current use at the time of cancer diagnosis). The validity of this assumption cannot be checked with freely available data, but marked change is unlikely in 3–5 years. For the exogenous hormones calculations there were no freely available data on prevalence of use by preparation type or duration of use, which necessitated a simplified (and arguably weaker) methodology in comparison to Parkin et al.^5^ The relative risks used in the calculations are not preparation or duration-specific and are from a UK population, so the distribution of preparation types and use durations in the exposure prevalence data are expected to be close to that in the relative risk data.

Calculations for ionising radiation may overestimate radon-attributable cases as radon prevalence at country level was taken from recent Public Health England data which focuses on high-radon areas rather than a random sample;^51^ however, it is unlikely that the magnitude of overestimation varies between countries.

### Expectations for future years’ PAFs in the UK

Tobacco smoking currently contributes by far the largest proportion of UK cancer cases attributable to risk factor exposure, and as prevalence of this behaviour is falling, so the tobacco PAF is expected to fall in future. This assumes that tobacco smoking prevalence will continue to fall in future, but this is not guaranteed; progress to date in this area is thanks to public health initiatives, including mass media cessation campaigns, Stop Smoking Services, smoke free legislation and plain packaging for tobacco products.^52^ Despite this, a wide disparity in smoking rates exists between different societal groups, for example, rates remain very high among those with mental health conditions.^53^ Some groups will need more support to quit so effective smoking cessation interventions should continue to be provided by the government and the NHS to maintain the current momentum and address health inequalities.^54^

Overweight and obesity contributes the second-highest proportion of attributable cases and prevalence of this risk factor is rising, so this PAF is expected to rise in future. Evidence for the impact of high BMI on cancer risk is still growing, so more cancer types could also be classified as having strong evidence for an association with BMI, which would also increase the PAF. The PAF gap between tobacco and overweight and obesity will shrink in future if current overweight and obesity prevalence trends continue. Current initiatives, including the UK Government’s Soft Drinks Industry Levy and Sugar Reduction programme, may slow the increase, but a more comprehensive approach as seen in tobacco may be necessary to significantly reduce prevalence.^55^ This should include recommendations made by Public Health England such as restrictions to the advertising of foods high in fat, sugar, and salt.^56^

Factors not included in these calculations may impact on PAFs by affecting the mix of cancer types in the all cancers combined total. Screening for bowel, cervical and breast cancer, and HPV vaccination, may reduce the proportion of cancer types which contribute a large number of preventable cases in the current calculations, reducing the overall PAF. Introduction of further screening programmes would also affect the overall PAFs.^57–59^ In addition, incidence could fall for some cancers in the future with more conservative testing practice—for example, if prostate cancer incidence falls with more conservative use of PSA testing in future, the proportion of non-risk-factor-attributable cancer cases in the all cancers combined total will be reduced, increasing the overall PAF.

## Conclusion

Known risk factors are responsible for a substantial proportion of UK cancer cases. Prevention efforts which focus on smoking and overweight and obesity are likely to have the largest population-level impact. Between-country variation likely reflects population demographics; deprived communities across the UK require additional support to reduce their cancer risk. Evidence from this study should be used to focus efforts on reducing the number and proportions of cancers attributable to preventable risk factors across the countries of the UK Department of Health.^20^

## Electronic supplementary material

Cancer attributable to risk factors, UK 2015 - Final Supplementary Material A-E

Cancer attributable to risk factors, UK 2015 - Final Supplementary Material F
