# Supplementary material for: The fraction of cancer attributable to modifiable risk factors in England, Wales, Scotland, Northern Ireland, and the United Kingdom in 2015
Source: Br J Cancer. 2018 Mar 23;118(8):1130–41. doi: 10.1038/s41416-018-0029-6 (PMC5931106; doi:10.1038/s41416-018-0029-6)
Supplement: Supplementary file 1 — Cancer attributable to risk factors, UK 2015 - Final Supplementary Material A-E [file 41416_2018_29_MOESM1_ESM.docx]

**The fraction of cancer attributable to modifiable risk factors in England, Wales, Scotland, Northern Ireland, and the United Kingdom in 2015**

**Supplementary Material A-E**

KF Brown^1*^, H Rumgay^1^, C Dunlop^1^, M Ryan^1^, F Quartly^1^, A Cox^1^, A Deas^2^, L Elliss-Brookes^3^, A Gavin^4^, L Hounsome^3^, D Huws^5^, N Ormiston-Smith^1^, J Shelton^1^, C White^5^, DM Parkin^6^.

^1^ Policy and Information Directorate, Cancer Research UK, The Angel Building, 407 St John Street, London EC1V 4AD; ^2^ NHS National Services Scotland, Information Services Division, Meridian Court, 5 Cadogan Street, Glasgow G2 6QE;

^3^ National Cancer Registration and Analysis Service, Public Health England, 2nd Floor, Skipton House, 80 London Road, London SE1 6LH;  ^4^ Northern Ireland Cancer Registry, Centre for Public Health, Queens University Belfast, Mulhouse Building, Grosvenor Road, Belfast BT12 6DP; ^5^ Welsh Cancer Intelligence and Surveillance Unit, Floor 5, Public Health Wales, Number 2 Capital Quarter, Tyndall Street, Cardiff CF10 4BZ; ^6^ Centre for Cancer Prevention, Wolfson Institute of Preventive Medicine, Queen Mary University of London, Charterhouse Square, London EC1M 6BQ

^*^ Correspondence: Dr Katrina Brown, [katrina.brown@cancer.org.uk](mailto:katrina.brown@cancer.org.uk) or [stats.team@cancer.org.uk](mailto:stats.team@cancer.org.uk).

[Supplementary Material A: Combinations of risk factor and cancer type included, with classification source 2](#_Toc502257558)

[Supplementary Material B: Search terms for identifying relative risks 4](#_Toc502257559)

[Supplementary Material C: Relative risk figures used 6](#_Toc502257560)

[Supplementary Material D: Summary prevalence of exposure to risk factors, by country and sex 9](#_Toc502257561)

[Supplementary Material E: Calculations on relative risk or exposure prevalence data 12](#_Toc502257562)

[References for Supplementary Material 16](#_Toc502257563)

Please see separate XLS file for Supplementary Material F: Results by country, risk factor and cancer type combinations.

# Supplementary Material A: Combinations of risk factor and cancer type included, with classification source

|  | **Oral cavity (C00-C06)** | **Nasopharynx (C11)** | **Pharynx (C09, C10, C12-C14)** | **Oesophagus (C15)** | **Stomach (C16)** | **Bowel (C18-C20)** | **Anus (C21)** | **Liver (C22)** | **Pancreas (C25)** | **Gallbladder (C23)** | **Sino-nasal (C30-C31)** | **Larynx (C32)** | **Lung (C33-C34)** | **Bone (C40-41)** | **Mesothelioma (C45)** | **Melanoma (C43)** | **Kaposi sarcoma (C46)** |
| --- | --- | --- | --- | --- | --- | --- | --- | --- | --- | --- | --- | --- | --- | --- | --- | --- | --- |
| **Tobacco** | I 12 | I 12 | I 12 | I 12 | I 12 | I 12 |  | I 12 | I 12 |  | I 12 | I 12 | I 12 |  |  |  |  |
| **Overweight and obesity** |  |  |  | I 16 | I 16 | I 16 |  | I 16 | I 16 | I 16 |  |  |  |  |  |  |  |
| **Radiation - UV** |  |  |  |  |  |  |  |  |  |  |  |  |  |  |  | I 12 |  |
| **Occupation** |  | I 04-17 |  |  | I 04-17 |  |  | I 04-17 |  | I 04-17 | I 04-17 | I 04-17 | I 04-17 |  | I 04-17 | I 04-17 |  |
| **Infections** | I 12 | I 12 | I 12 |  | I 12 |  | I 12 | I 12 |  |  |  |  |  |  |  |  | I 12 |
| **Alcohol** | I 12 |  | I 12 | W 16 |  | I 12 |  | W 15 |  |  |  | I 12 |  |  |  |  |  |
| **Fibre** |  |  |  |  |  | W 17 |  |  |  |  |  |  |  |  |  |  |  |
| **Radiation - ionising** | I 12 |  |  | I 12 | I 12 | I 12 |  |  |  |  | I 12 |  |  | I 12 |  | I 12 |  |
| **Processed meat** |  |  |  |  |  | W 17 |  |  |  |  |  |  |  |  |  |  |  |
| **Air pollution** |  |  |  |  |  |  |  |  |  |  |  |  | I 16 |  |  |  |  |
| **Not breastfeeding** |  |  |  |  |  |  |  |  |  |  |  |  |  |  |  |  |  |
| **Insufficient physical activity** |  |  |  |  |  | W 17 |  |  |  |  |  |  |  |  |  |  |  |
| **Post-menopausal hormones** |  |  |  |  |  | I 12 |  |  |  |  |  |  |  |  |  |  |  |
| **Oral contraceptives** |  |  |  |  |  | I 12 |  |  |  |  |  |  |  |  |  |  |  |
| I = International Agency for Research on Cancer (IARC) classification. W = World cancer Research Fund (WCRF) classification. Numbers are the year in which the classification was published. | | | | | | | | | | | | | | | | | |

|  | **Breast (C50)** | **Vulva (C51)** | **Vagina (C52)** | **Cervix (C53)** | **Uterus (C54-C55)** | **Ovary (C56)** | **Penis (C60)** | **Bladder (C67)** | **Kidney (C64-C66, C68)** | **Eye (C69)** | **Thyroid (C73)** | **Myeloma (C90)** | **Hodgkin lymphoma (C81)** | **NHL (C82-C85, C96)** | **Leukaemia (C91-C95)** | **Brain and CNS (C70-C72)** |
| --- | --- | --- | --- | --- | --- | --- | --- | --- | --- | --- | --- | --- | --- | --- | --- | --- |
| **Tobacco** |  |  |  | I 12 |  | I 12 |  | I 12 | I 12 |  |  |  |  |  | I 12 |  |
| **Overweight and obesity** | I 16 |  |  |  | I 16 | I 16 |  |  | I 16 |  | I 16 | I 16 |  |  |  | I 16 |
| **Radiation - UV** |  |  |  |  |  |  |  |  |  |  |  |  |  |  |  |  |
| **Occupation** |  |  |  |  |  | I 04-17 |  | I 04-17 | I 04-17 | I 04-17 |  |  |  |  | I 04-17 |  |
| **Infections** |  | I 12 | I 12 | I 12 |  |  | I 12 |  |  | I 12 |  |  | I 12 | I 12 |  |  |
| **Alcohol** | W 17 |  |  |  |  |  |  |  |  |  |  |  |  |  |  |  |
| **Fibre** |  |  |  |  |  |  |  |  |  |  |  |  |  |  |  |  |
| **Radiation - ionising** | I 12 |  |  |  |  |  |  | I 12 | I 12 |  | I 12 |  |  |  | I 12 | I 12 |
| **Processed meat** |  |  |  |  |  |  |  |  |  |  |  |  |  |  |  |  |
| **Air pollution** |  |  |  |  |  |  |  |  |  |  |  |  |  |  |  |  |
| **Not breastfeeding** | W 10 |  |  |  |  |  |  |  |  |  |  |  |  |  |  |  |
| **Insufficient physical activity** |  |  |  |  |  |  |  |  |  |  |  |  |  |  |  |  |
| **Post-menopausal hormones** | I 12 |  |  |  | I 12 | I 12 |  |  |  |  |  |  |  |  |  |  |
| **Oral contraceptives** | I 12 |  |  | I 12 | I 12 | I 12 |  |  |  |  |  |  |  |  |  |  |
| I = International Agency for Research on Cancer (IARC) classification. W = World cancer Research Fund (WCRF) classification. Numbers are the year in which the classification was published. | | | | | | | | | | | | | | | | |

# Supplementary Material B: Search terms for identifying relative risks

Combinations of cancer type and risk factor search strings were made using AND. Searches were made in PubMed, and were supplemented using Google Scholar and scrutiny of reference lists in other relevant papers.

| **Cancer type** | **Search string** |
| --- | --- |
| Melanoma skin cancer | melanoma OR skin AND (cancer OR tumour) |
| Oral cavity cancer | (oral OR mouth) AND (cancer OR tumour) |
| Nasopharyngeal cancer | (nasopharynx OR nasopharyngeal) AND (cancer OR tumour) |
| Pharyngeal cancer | (oropharynx OR oropharyngeal OR pharynx OR pharyngeal) AND (cancer OR tumour) |
| Oesophageal cancer | (oesophagus OR oesophageal) AND (cancer OR adenocarcinoma OR squamous cell AND (cancer OR tumour) |
| Stomach cancer | (stomach OR gastric OR cardia) AND (cancer OR adenocarcinoma OR tumour) |
| Bowel cancer | (colorectum OR colorectal OR colon OR rectum OR rectal OR bowel) AND (cancer OR tumour) |
| Anal cancer | (anus OR anal) AND (cancer OR tumour) |
| Liver cancer | (liver OR hepatic OR hepatocellular) AND (cancer OR carcinoma OR tumour) |
| Pancreatic cancer | (pancreas OR pancreatic) AND (cancer OR adenocarcinoma OR tumour) |
| Gallbladder cancer | Gallbladder AND (cancer OR tumour) |
| Laryngeal cancer | (larynx OR laryngeal) AND (cancer OR tumour) |
| Lung cancer | lung AND (cancer OR adenocarcinoma OR squamous cell carcinoma OR tumour) |
| Mesothelioma | mesothelioma |
| Kaposi sarcoma | kaposi sarcoma |
| Breast cancer | breast AND (cancer OR carcinoma OR tumour) |
| Vulval cancer | (vulva OR vulval) AND (cancer OR tumour) |
| Vaginal cancer | (vagina OR vaginal) AND (cancer OR tumour) |
| Cervical cancer | (cervix OR cervical) AND (cancer OR tumour) |
| Uterine cancer | (uterus OR uterine OR endometrium OR endometrial) AND (cancer OR carcinoma OR tumour) |
| Ovarian cancer | (ovary OR ovarian) AND (cancer OR carcinoma OR tumour) |
| Penile cancer | (penis OR penile) AND (cancer OR carcinoma OR tumour) |
| Prostate cancer | (prostate OR prostatic) AND (cancer OR carcinoma OR tumour) |
| Bladder cancer | (bladder OR urothelium OR urothelial) AND (cancer OR carcinoma OR tumour) |
| Kidney cancer | (kidney OR renal OR renal cell) AND (cancer OR carcinoma OR tumour) |
| Thyroid cancer | thyroid (cancer OR tumour) |
| Myeloma | myeloma |
| Hodgkin lymphoma | Hodgkin lymphoma OR Hodgkin’s disease |
| Non-Hodgkin lymphoma | non-Hodgkin lymphoma |
| Leukaemia | leukaemia OR leukemia |
| Brain and other central nervous system tumours | (brain OR nervous system OR spinal cord OR glioma OR meningioma) AND (cancer OR tumour) |

| **Risk factor** | **Search string** |
| --- | --- |
| Tobacco | tobacco OR cigarette OR smoking OR environmental tobacco smoke OR secondhand smoke |
| Overweight and obesity | weight OR BMI OR body mass index OR obesity OR obese OR overweight OR adiposity OR body size |
| Radiation - UV | (ultraviolet OR UV OR solar) AND radiation |
| Occupation | Not sought – used Rushton et al 2010 |
| Infections | hepatitis B virus OR HBV  hepatitis C virus OR HCV  human papillomavirus OR HPV  human immunodeficiency virus OR HIV OR acquired immune deficiency syndrome OR AIDS  Helicobacter pylori OR H. pylori  Epstein Barr virus OR EBV  Kaposi sarcoma herpesvirus OR KSHV OR human herpesvirus 8 OR HHV8 |
| Alcohol | alcohol OR alcoholic OR ethanol |
| Insufficient fibre | fibre OR fiber |
| Radiation - ionising | radon  x-ray  nuclear medicine OR radio-isotopes therapy  radiotherapy |
| Processed meat | Meat OR bacon OR ham OR sausages OR jerky OR salami OR cured OR salted |
| Air pollution | (air OR environment OR outdoor) AND pollution |
| Not breastfeeding | breastfeeding OR breastfed OR lactation |
| Insufficient physical activity | physical OR activity OR exercise OR physically active OR sedentary |
| Post-menopausal hormones | hormone replacement therapy OR ((menopausal OR menopause) AND hormone therapy) OR |
| Oral contraceptives | (oral AND (contraceptive OR contraception)) OR birth control pill |

# Supplementary Material C: Relative risk figures used

| **Risk factor^a^** | **Cancer type** | | | | | | | | | | | | | | | | | | | | | | | | |
| --- | --- | --- | --- | --- | --- | --- | --- | --- | --- | --- | --- | --- | --- | --- | --- | --- | --- | --- | --- | --- | --- | --- | --- | --- | --- |
|  | **Lung** | **Oral cavity^i^** | **Nasopharynx** | **Pharynx** | **Oesophageal AC** | **Oesophageal SCC** | **Stomach^e^** | **Liver** | **Pancreas** | **Colon** | **Rectum** | **Larynx** | **Cervix** | **Ovary^b^** | **Bladder** | **Kidney** | **Leukaemia^c^** | **Breast^d^** | **Uterus** | **Gallbladder** | **Brain^f^** | **Thyroid** | **Myeloma** | **Non-Hodgkin Lymphoma^g^** | **Other cancer types^h^** |
|  | | | | | | | | | | | | | | | | | | | | | | | | | |
| **Tobacco (cigarette) smoking ^[[1]](#endnote-1)^ ^[[2]](#endnote-2)^ ^[[3]](#endnote-3)^ ^[[4]](#endnote-4)^ ^[[5]](#endnote-5)^ ^[[6]](#endnote-6)^ ^[[7]](#endnote-7)^ ^[[8]](#endnote-8)^ ^[[9]](#endnote-9)^ ^[[10]](#endnote-10)^ ^[[11]](#endnote-11)^ ^[[12]](#endnote-12)^ ^[[13]](#endnote-13)^ ^[[14]](#endnote-14)^ ^[[15]](#endnote-15)^** | | | | | | | | | | | | | | | | | | | | | | | | | |
| *Current vs never* | | | | | | | | | | | | | | | | | | | | | | | | | |
| Males | 8.96 | 1.91 | 1.95 | 3.43 | 2.32 | 4.21 | 1.62 | 1.61 | 2.20 | 1.11 | 1.44 | 7.01 |  |  | 3.44 | 1.35 | 1.47 |  |  |  |  |  |  |  |  |
| Females | 8.96 | 1.91 | 1.95 | 3.43 | 2.32 | 4.21 | 1.20 | 1.86 | 2.20 | 1.11 | 1.44 | 7.01 | 1.83 | 1.49 | 3.56 | 1.35 | 1.47 |  |  |  |  |  |  |  |  |
| *Former vs never* | | | | | | | | | | | | | | | | | | | | | | | | | |
| Males | 3.85 | 1 | 1.39 | 1 | 1.62 | 2.18 | 1.34 | 1.47 | 1.17 | 1.15 | 1.11 | 2.37 |  |  | 1.92 | 1.22 | 1 |  |  |  |  |  |  |  |  |
| Females | 3.85 | 1 | 1.39 | 1 | 1.62 | 2.18 | 1 | 1 | 1.17 | 1.15 | 1.11 | 2.37 | 1.26 | 1 | 2.04 | 1.22 | 1 |  |  |  |  |  |  |  |  |
| *Secondhand exposed vs unexposed* | | | | | | | | | | | | | | | | | | | | | | | | | |
| Males | 1.23 |  |  |  |  |  |  |  |  |  |  |  |  |  |  |  |  |  |  |  |  |  |  |  |  |
| Females | 1.37 |  |  |  |  |  |  |  |  |  |  |  |  |  |  |  |  |  |  |  |  |  |  |  |  |
|  | | | | | | | | | | | | | | | | | | | | | | | | | |
| **Overweight and obesity ^[[16]](#endnote-16)^ ^[[17]](#endnote-17)^ ^[[18]](#endnote-18)^ ^[[19]](#endnote-19)^ ^[[20]](#endnote-20)^ ^[[21]](#endnote-21)^ ^[[22]](#endnote-22)^ ^[[23]](#endnote-23)^ ^[[24]](#endnote-24)^ ^[[25]](#endnote-25)^ ^[[26]](#endnote-26)^ ^[[27]](#endnote-27)^ ^[[28]](#endnote-28)^** | | | | | | | | | | | | | | | | | | | | | | | | | |
| *Overweight vs healthy weight (BMI 25<30 vs BMI 18.5<25)* | | | | | | | | | | | | | | | | | | | | | | | | | |
| Males |  |  |  |  | 1.87 |  | 1.22 | 1.18 | 1.15 | 1.17 | 1.17 |  |  |  |  | 1.22 |  |  |  | 1 | 1 | 1.1 | 1.17 |  |  |
| Females |  |  |  |  | 1.87 |  | 1.22 | 1.18 | 1.12 | 1.07 | 1.07 |  |  | 1.08 |  | 1.38 |  | 1.13 | 1.34 | 1.22 | 1 | 1.1 | 1.12 |  |  |
| *Obese vs healthy weight (BMI 30+ vs BMI 18.5<25)* | | | | | | | | | | | | | | | | | | | | | | | | | |
| Males |  |  |  |  | 2.73 |  | 1.61 | 1.83 | 1.20 | 1.38 | 1.38 |  |  |  |  | 1.63 |  |  |  | 1.54 | 1 | 1.27 | 1.23 |  |  |
| Females |  |  |  |  | 2.73 |  | 1.61 | 1.83 | 1.15 | 1.17 | 1.17 |  |  | 1.11 |  | 1.95 |  | 1.20 | 2.54 | 1.75 | 1.60 | 1.27 | 1.15 |  |  |
|  | | | | | | | | | | | | | | | | | | | | | | | | | |
| **Infections** | | | | | | | | | | | | | | | | | | | | | | | | | |
| *Helicobacter pylori (H. pylori) ^[[29]](#endnote-29)^* | | | | | | | | | | | | | | | | | | | | | | | | | |
| Persons |  |  |  |  |  |  | 5.90 |  |  |  |  |  |  |  |  |  |  |  |  |  |  |  |  | 6.30 |  |
| *Hep B* *^[[30]](#endnote-30)^* | | | | | | | | | | | | | | | | | | | | | | | | | |
| Persons |  |  |  |  |  |  |  | 20.3 |  |  |  |  |  |  |  |  |  |  |  |  |  |  |  |  |  |
| *Hep C* 38 *^[[31]](#endnote-31)^* | | | | | | | | | | | | | | | | | | | | | | | | | |
| Persons |  |  |  |  |  |  |  | 23.8 |  |  |  |  |  |  |  |  |  |  |  |  |  |  |  | 2.03 |  |
| *HIV ^[[32]](#endnote-32)^ ^[[33]](#endnote-33)^* | | | | | | | | | | | | | | | | | | | | | | | | | |
| Persons |  |  |  |  |  |  |  |  |  |  |  |  |  |  |  |  |  |  |  |  |  |  |  | 10.6 | 8.06 |
|  | | | | | | | | | | | | | | | | | | | | | | | | | |
|  | | | | | | | | | | | | | | | | | | | | | | | | | |
| **Alcohol** **^[[34]](#endnote-34)^** | | | | | | | | | | | | | | | | | | | | | | | | | |
| *Light (median daily ≤12.5g ethanol) vs never* | | | | | | | | | | | | | | | | | | | | | | | | | |
| Persons |  | 1 |  | 1 |  | 1.34 |  | 1 |  | 1 | 1 | 1 |  |  |  |  |  | 1.04 |  |  |  |  |  |  |  |
| *Moderate (median daily 12.5-50g ethanol) vs never* | | | | | | | | | | | | | | | | | | | | | | | | | |
| Persons |  | 1.81 |  | 1.81 |  | 2.56 |  | 1 |  | 1.17 | 1.17 | 1.49 |  |  |  |  |  | 1.23 |  |  |  |  |  |  |  |
| *Heavy (median daily 50g+ ethanol) vs never* | | | | | | | | | | | | | | | | | | | | | | | | | |
| Persons |  | 5.07 |  | 5.07 |  | 5.45 |  | 2.16 |  | 1.33 | 1.33 | 2.39 |  |  |  |  |  | 1.60 |  |  |  |  |  |  |  |
|  |  |  |  |  |  |  |  |  |  |  |  |  |  |  |  |  |  |  |  |  |  |  |  |  |  |
| **Fibre (per 1g deficit per day) ^[[35]](#endnote-35)^** | | | | | | | | | | | | | | | | | | | | | | | | | |
| Persons |  |  |  |  |  |  |  |  |  | 1.03 | 1.03 |  |  |  |  |  |  |  |  |  |  |  |  |  |  |
|  |  |  |  |  |  |  |  |  |  |  |  |  |  |  |  |  |  |  |  |  |  |  |  |  |  |
| **Ionising radiation** | | | | | | | | | | | | | | | | | | | | | | | | | |
| *Background radiation (cosmic, gamma, internal, per Sv)^l^* **^[[36]](#endnote-36)^** | | | | | | | | | | | | | | | | | | | | | | | | | |
| Persons | 1.02 | 1.03 |  |  | 1.02 |  | 1.01 |  |  | 1.02 | 1 |  |  |  | 1.02 | 1 | <1.2 | 1.02 |  |  | 1.02 | 1.02 |  |  | 1.03 |
| *Radon (per 100 Bq/m^3^)* ^[[37]](#endnote-37)^ | | | | | | | | | | | | | | | | | | | | | | | | | |
| Persons | 1.16 |  |  |  |  |  |  |  |  |  |  |  |  |  |  |  |  |  |  |  |  |  |  |  |  |
|  |  |  |  |  |  |  |  |  |  |  |  |  |  |  |  |  |  |  |  |  |  |  |  |  |  |
| **Processed meat (per 50g per day) ^[[38]](#endnote-38)^** | | | | | | | | | | | | | | | | | | | | | | | | | |
| Persons |  |  |  |  |  |  |  |  |  | 1.13 | 1.13 |  |  |  |  |  |  |  |  |  |  |  |  |  |  |
|  |  |  |  |  |  |  |  |  |  |  |  |  |  |  |  |  |  |  |  |  |  |  |  |  |  |
| **Air pollution ^[[39]](#endnote-39)^** | | | | | | | | | | | | | | | | | | | | | | | | | |
| *Anthropogenic PM_2.5_, per µg m^3^* | | | | | | | | | | | | | | | | | | | | | | | | | |
| Persons | 1.09 |  |  |  |  |  |  |  |  |  |  |  |  |  |  |  |  |  |  |  |  |  |  |  |  |
| *Anthropogenic PM_10_, per µg m^3^* | | | | | | | | | | | | | | | | | | | | | | | | | |
| Persons | 1 |  |  |  |  |  |  |  |  |  |  |  |  |  |  |  |  |  |  |  |  |  |  |  |  |
|  |  |  |  |  |  |  |  |  |  |  |  |  |  |  |  |  |  |  |  |  |  |  |  |  |  |
| **Breastfeeding (never vs ever) ^[[40]](#endnote-40)^** | | | | | | | | | | | | | | | | | | | | | | | | | |
| Females |  |  |  |  |  |  |  |  |  |  |  |  |  |  |  |  |  | 1.08 |  |  |  |  |  |  |  |
|  |  |  |  |  |  |  |  |  |  |  |  |  |  |  |  |  |  |  |  |  |  |  |  |  |  |
|  |  |  |  |  |  |  |  |  |  |  |  |  |  |  |  |  |  |  |  |  |  |  |  |  |  |
|  | | | | | | | | | | | | | | | | | | | | | | | | | |
| **Physical activity (***600-3999 vs <600 MET-minutes per week ^j^***) ^[[41]](#endnote-41)^** | | | | | | | | | | | | | | | | | | | | | | | | | |
| Persons |  |  |  |  |  |  |  |  |  | 0.90 |  |  |  |  |  |  |  |  |  |  |  |  |  |  |  |
|  | | | | | | | | | | | | | | | | | | | | | | | | | |
| **Post-menopausal hormones ^[[42]](#endnote-42)^ ^[[43]](#endnote-43)^** | | | | | | | | | | | | | | | | | | | | | | | | | |
| Ex- (5+ years use, 5+ years since use) vs never-users | | | | | | | | | | | | | | | | | | | | | | | | | |
| Females |  |  |  |  |  |  |  |  |  |  |  |  |  | 1.10 |  |  |  | 1 |  |  |  |  |  |  |  |
| *Current (5+ years use) vs never-users* | | | | | | | | | | | | | | | | | | | | | | | | | |
| Females |  |  |  |  |  |  |  |  |  |  |  |  |  | 1.41 |  |  |  | 1.66 |  |  |  |  |  |  |  |
|  | | | | | | | | | | | | | | | | | | | | | | | | | |
| **Oral contraceptives (current- vs never-users ^k^) ^[[44]](#endnote-44)^ ^[[45]](#endnote-45)^** | | | | | | | | | | | | | | | | | | | | | | | | | |
| Females |  |  |  |  |  |  |  |  |  |  |  |  | 1.90 |  |  |  |  | 1.21 |  |  |  |  |  |  |  |
|  | | | | | | | | | | | | | | | | | | | | | | | | | |
| Key to superscript notes overleaf | | | | | | | | | | | | | | | | | | | | | | | | | |
| *a Relative risks obtained only for cancer type-risk factor combinations classified by IARC as ‘sufficient’ or WCRF as ‘convincing’; blank cells indicate no RR was sought as the combination is not classified as above. RR = 1 if cancer type-risk factor association is not significant in the source evidence chosen. No RRs shown for Epstein-Barr virus, Human Papillomavirus (HPV), Kaposi Sarcoma Herpesvirus/Human Herpesvirus 8 (KSHV/HHV8), and diagnostic radiation, because for these factors PAFs were identified in the literature rather than being calculated within this project* | | | | | | | | | | | | | | | | | | | | | | | | | |
| *b Mucinous ovarian cancer only for tobacco (cigarette) smoking* | | | | | | | | | | | | | | | | | | | | | | | | | |
| *c Acute myeloid leukaemia only for tobacco (cigarette) smoking, all leukaemia excluding chronic lymphocytic for ionising radiation (RR varies with age, dose, sex, age at and time since exposure so RR given is upper bound)* | | | | | | | | | | | | | | | | | | | | | | | | | |
| *d Postmenopausal breast cancer only for overweight and obesity, female breast cancer only for alcohol* | | | | | | | | | | | | | | | | | | | | | | | | | |
| *e Gastric cardia cancer only for overweight and obesity, non-cardia only for H. pylori* | | | | | | | | | | | | | | | | | | | | | | | | | |
| *f Meningioma only for overweight and obesity; brain, other central nervous system and intracranial tumours (malignant, benign and uncertain or unknown behaviour) for ionising radiation* | | | | | | | | | | | | | | | | | | | | | | | | | |
| *g Mucosa-associated lymphoid tissue (MALT) lymphoma only for H. pylori* | | | | | | | | | | | | | | | | | | | | | | | | | |
| *h Conjunctiva for HIV; bone and ‘all other solid cancers’ for ionising radiation (background radiation)* | | | | | | | | | | | | | | | | | | | | | | | | | |
| *i Salivary gland for ionising radiation (background radiation)* | | | | | | | | | | | | | | | | | | | | | | | | | |
| *j RR = 0.0022* | | | | | | | | | | | | | | | | | | | | | | | | | |
| *k 0-5 years since last use (breast), ‘current’ and 5+ years use (cervix)* | | | | | | | | | | | | | | | | | | | | | | | | | |
| *l Relative risks converted from percent per Sievert and used in calculations as excess relative risk per mSv, e.g. risks shown in this table are for much higher exposure levels than seen in UK population* | | | | | | | | | | | | | | | | | | | | | | | | | |

# Supplementary Material D: Summary prevalence of exposure to risk factors, by country and sex

| **Risk factor** | **England** | **Scotland** | **Wales** | **Northern Ireland** | **Optimum exposure** |
| --- | --- | --- | --- | --- | --- |
| **Tobacco (cigarette) smoking (%)** **^[[46]](#endnote-46)^** **^[[47]](#endnote-47)^** **^[[48]](#endnote-48)^** **^[[49]](#endnote-49)^** | | | | | |
| Data years | 2005 | 2005 | 2004/05 | 2004/05 | Nil |
| *Current* | | | | | |
| Males 16+ | 27 | 28 | 29 | 27 |  |
| Females 16+ | 24 | 24 | 30 | 25 |  |
| *Former* | | | | | |
| Males 16+ | 28 | 27 | 26 | 23 |  |
| Females 16+ | 20 | 21 | 24 | 13 |  |
| **Exposure to secondhand smoke (%) ^a^ 46 47 48 49** | | | | | |
| Data years | 2005 | 2003 | 2004 | GB average | Nil |
| *Some exposure* | | | | | |
| Males 16-75 | 58 | 63 | 73 | 65 |  |
| Females 16-75 | 48 | 57 | 67 | 57 |  |
| **Overweight and obesity (%)** **46 47 48 ^[[50]](#endnote-50)^** | | | | | |
| Data years | 2005 | 2005 | 2004/05 | 2005/06 | BMI 18<25 |
| *Overweight (BMI 25<30)* |  |  |  |  |  |
| Males 16+ | 43 | 41 | 42 | 39 |  |
| Females 16+ | 32 | 31 | 32 | 30 |  |
| *Obese (BMI 30+)* |  |  |  |  |  |
| Males 16+ | 22 | 22 | 18 | 25 |  |
| Females 16+ | 24 | 23 | 18 | 23 |  |
| **Occupation (industry sectors with highest PAFs)% of total jobs)** **^[[51]](#endnote-51)^** | | | | | |
| Data years | 1982 | 1982 | 1982 | 1982 | Nil |
| *Manufacturing* | 23 | 21 | 21 | 22 |  |
| *Construction* | 5 | 7 | 6 | 6 |  |
| *Transport and storage* | 5 | 5 | 4 | 3 |  |
| **Infections (%)** | | | | | |
| Data years | 2005 | 2005 | 2005 | 2005 | Nil |
| *H. pylori ^f^* ^^[[52]](#endnote-52)^^ ^^[[53]](#endnote-53)^^ ^^[[54]](#endnote-54)^^ | 17 | 61 | 17 | 57 |  |
| *Hep ^B^* ^^[[55]](#endnote-55)^^ | 0.5 | 0.5 | 0.5 | 0.5 |  |
| *Hep C ^g^* ^^[[56]](#endnote-56)^^ ^^[[57]](#endnote-57)^^ ^^[[58]](#endnote-58)^^ ^^[[59]](#endnote-59)^^ | 0.4 | 0.7 | 0.4 | 0.2 |  |
| *HIV* ^^[[60]](#endnote-60)^^ ^^[[61]](#endnote-61)^^ |  |  |  |  |  |
| Males 15-59 | 0.22 | 0.01 | 0 | 0 |  |
| Males 60+ | 0.02 | 0 | 0 | 0 |  |
| Females 15-59 | 0.01 | 0.01 | 0 | 0 |  |
| Females 60+ | 0.02 | 0 | 0 | 0 |  |
| **Alcohol drinking (%) ^[[62]](#endnote-62)^** | | | | | |
| Data years | 2005 | 2005 | 2005 | GB average | Nil |
| *Light (median daily intake ≤12.5g ethanol)* | | | | | |
| Males 16+ | 44 | 42 | 45 | 44 |  |
| Females 16+ | 54 | 58 | 57 | 56 |  |
| **Risk factor** | **England** | **Scotland** | **Wales** | **Northern Ireland** | **Optimum exposure** |
| *Moderate (median daily intake 12.5-50g ethanol)* | | | | | |
| Males 16+ | 34 | 35 | 38 | 36 |  |
| Females 16+ | 26 | 24 | 24 | 25 |  |
| *Heavy (median daily intake 50g+ ethanol)* | | | | | |
| Males 16+ | 12 | 11 | 10 | 11 |  |
| Females 16+ | 2 | 1 | 2 | 2 |  |
| **Fibre (g per day) ^d^** 51 52 53 54 | | | | | |
| Data years | 2000/01 | 2000/01 | 2000/01 | 2000/01 | 30g/day |
| Males 19+ | 20 | 19 | 19 | 18 |  |
| Females 19+ | 16 | 15 | 16 | 15 |  |
| **Ionising radiation (average mSv per year) ^[[63]](#endnote-63)^** | | | | | |
| Data years | 2010 | 2010 | 2010 | 2010 | Nil |
| *Background radiation ^h^* | 0.94 | 0.99 | 0.95 | 0.94 |  |
| *Radon* | 1.49 | 0.84 | 1.9 | 1.23 |  |
| **Processed meat (g per day) ^c^** ^[[64]](#endnote-64)^ ^[[65]](#endnote-65)^ ^[[66]](#endnote-66)^ ^[[67]](#endnote-67)^ | | | | | |
| Data years | 2000/01 | 2000/01 | 2000/01 | 2000/01 | Nil |
| Males 19+ | 74 | 77 | 68 | 80 |  |
| Females 19+ | 37 | 36 | 34 | 42 |  |
| **Air pollution (mean annual concentration of anthropogenic PM_2.5_ µg m^3^) ^[[68]](#endnote-68)^** | | | | | |
| Data years | 2010 | 2010 | 2010 | 2010 | Nil |
| Persons | 9.9 | 6.8 | 7.5 | 6.9 |  |
| **Breastfeeding (% never breastfed) ^[[69]](#endnote-69)^ ^[[70]](#endnote-70)^ ^[[71]](#endnote-71)^ ^[[72]](#endnote-72)^ ^[[73]](#endnote-73)^ ^[[74]](#endnote-74)^ ^[[75]](#endnote-75)^ ^[[76]](#endnote-76)^** | | | | | |
| Data years | 2016 | 2016 | 2016 | 2016 | Ever-br’stfed |
| Females 30-89 | 52 | 58 | 52 | 66 |  |
| **Physical activity (% achieving 150+ minutes moderate physical activity per week) 46 47 48 55** | | | | | |
| Data years | 2005 | 2005 | 2004/05 | 2005/06 | 150+ mins/week |
| Males 16+ | 39 | 43 | 36 | 33 |  |
| Females 16+ | 27 | 31 | 23 | 28 |  |
| **Post-menopausal hormones (%) ^e^** **^[[77]](#endnote-77)^** 57 | | | | | |
| Data years | 2010-12 | 2010-12 | 2010-12 | GB average | Nil |
| *Current use* | | | | | |
| Females 16-74 | 2 | 2 | 3 | 2 |  |
| Females 75+ | 0 | 0 | 0 | 0 |  |
| *Past use* | | | | | |
| Females 16-74 | 10 | 10 | 10 | 10 |  |
| Females 75+ | 22 | 25 | 19 | 22 |  |
| **Oral contraceptives (%)** 56 **^[[78]](#endnote-78)^** | | | | | |
| Data years | 2010-12 | 2010-12 | 2010-12 | ROI 2010 | Nil |
| *Current use (in last year)* | | | | | |
| Females 16-74 | 0-41 | 0-44 | 0-44 | 1-61 |  |
| Females 75+ | 0 | 0 | 0 | 0 |  |
|  | | | | | |
| *a Responded anything other than ‘never’ when asked ‘how many hours are you exposed to other people’s smoke’* | | | | | |
| *b Beef, veal and dishes; lamb and dishes; pork and dishes; liver, liver products and dishes* | | | | | |
| *c Bacon and ham; burgers and kebabs; sausages; meat pies and pastries; other meat and meat products* | | | | | |
| *d Data were provided as non-starch polysaccharides (NSP) grams per day and converted to fibre assuming 1g NSP = 1.28g fibre* | | | | | |
| *e specific postmenopausal hormone preparation not reported in survey data* | | | | | |
| *f H. pylori data from 1996 for England and Wales, 1992 for Scotland, 1986-87 for Northern Ireland* | | | | | |
| *g England is figure for white/other ethnicity non-IDUs only; from the cited paper* | | | | | |
| *h Cosmic, gamma, internal* | | | | | |

# Supplementary Material E: Calculations on relative risk or exposure prevalence data

*Tobacco smoking*

PAFs were calculated for 2015 and 2010, with the 2010 calculations to afford comparison with Parkin et al. The same RRs were used for both 2015 calculations. Both calculations used survey-reported smoking prevalence.46 47 48 49

*Secondhand smoke*

Data were available only for England, Scotland and Wales so the averages of these countries were used for the Northern Ireland figures. Scotland data on exposure to other people’s smoke were only collected in 2003 and 2008, but the Scotland public smoking ban came into force in 2006,^[[79]](#endnote-79)^ so a linear trend was assumed unlikely and the 2003 data were used in the analysis.

*Overweight and obesity*

Scotland data on body mass index were collected only in 2003 and 2008, so 2005 data were imputed assuming a linear trend between those two survey years.

*UV radiation*

UV PAFs were calculated using ratios of expected (in less UV-unexposed persons, and UV-unrelated melanoma morphologies) versus observed (in typically UV-exposed persons, and UV-related melanoma morphologies) melanoma skin cancer cases. Less-UV exposed was operationalised in several ways, in line with previous work,^[[80]](#endnote-80)^ and the final PAF was an average of the PAFs obtained using each of these definitions. Less UV-exposed persons were those in the 1918 birth cohort, whose expected melanoma skin cancer rates were calculated using an age-period-cohort model. Acral lentiginous melanoma was considered UV-unrelated.89

*Occupation*

Recalculating PAFs by cancer type for each UK country was not possible with publicly available occupation data, so the all cancers combined occupation PAF from the original UK attributable cancers project was converted to country-specific all cancers combined occupation PAFs, with no further breakdown by cancer type.**^[[81]](#endnote-81)^** The breakdown of total jobs in 1982 by industry group was calculated for each country and for Great Britain. 69 The ratio of those percentages (e.g. Scotland:Great Britain) was applied to the Great Britain all cancers combined PAF (persons) for each industry, to obtain PAFs by industry by country. For example, manufacturing was 23% of total jobs in Great Britain, and 21% of total jobs in Scotland, so the Scotland PAF for manufacturing was 0.92 × the Great Britain PAF for manufacturing. Within these calculations non-melanoma skin cancer (NMSC) cases and shiftwork-attributable cancer cases were excluded; these were included in the original PAFs by industry but NMSC registration is insufficiently complete to include in PAFs,^[[82]](#endnote-82)^ and there is not sufficient evidence that shiftwork causes cancer in humans, according to IARC.^[[83]](#endnote-83)^ PAFs by industry and country were summed cumulatively to obtain PAFs for all industries and all cancers combined, by country. To obtain male and female PAFs, ratios (male to persons and female to persons) from the original UK attributable cancers project report were applied to the persons PAF.90

*Infections*

Data on *H. pylori* prevalence were available only for a subset of age bands for the devolved nations compared with England, so missing values were imputed by applying the average percentage difference between all observed age points to the age point at each end of the observed range, and then applying that same percentage change to those imputed age points, and so on. Data on Hepatitis B prevalence was available only for the UK overall, so the same prevalence rate was assumed to apply across all UK countries and the age breakdown for hepatitis C was applied to these data, because the risk factors are similar for both infections.^[[84]](#endnote-84)^ Data on hepatitis C prevalence in devolved nations were extrapolated from England data, by applying the age breakdown observed in the England data to the total population reported prevalence for Scotland and Wales, and by applying to the England data a conversion factor derived from first-time blood donors for Northern Ireland. Data on HIV prevalence were available for the UK only in the most appropriate data year, so that UK prevalence was broken down by country according to the percentages of UK total new HIV diagnoses in 2005 contributed by each UK country.

*Alcohol*

Prevalence of alcohol use was provided in units per week but the RRs were defined as grams of ethanol per day. Units per week was converted to grams of ethanol per day (units per week divided by 7, multiplied by 8g ethanol per unit),^[[85]](#endnote-85)^ and this was mapped to low, moderate and high daily alcohol consumption as defined in grams of ethanol per day in the source of the alcohol RRs. Data were available only for England, Scotland and Wales so the averages of these countries were used for the Northern Ireland figures.

*Processed meat and fibre*

Prevalence of processed meat and fibre consumption was provided for Great Britain only, in the survey period most suited to the ten-year lag (2000/01). To obtain UK country breakdowns, ratios of processed meat and fibre intake in the UK overall versus each UK country were calculated from the same survey in a more recent data period (2008-12),^[[86]](#endnote-86)^ and applied to the Great Britain figures from 2000/01.

*Ionising radiation*

Data on radon exposure were provided in average millisieverts (mSv) per year but the RRs were defined in becquerels per metre cubed (Bq m^3^). mSv per year were converted to Bq m^3^ assuming that exposure to an average indoor radon concentration in air of 20 Bq m^3^ results in an effective dose of about 1 mSv per year.^[[87]](#endnote-87)^ Data on the prevalence of radiotherapy use by cancer type was obtained from the original UK attributable cancers project report,^[[88]](#endnote-88)^ but the prevalence of cancer survivors was updated.^[[89]](#endnote-89)^ Data on background radiation were obtained for 2010, allowing a 5-year lag against incidence as in the original attributable cancers project.

*Breastfeeding*

Prevalence of ever-breastfeeding was calculated by identifying the median year in which each birth cohort had their first baby, and the percentage of women giving birth in that year who breastfed initially, then applying that percentage to the percentage of women who were parous by age 45. Data on median year of first birth (calculated from median age at first birth per cohort) was available for birth cohorts 1920-1986 for England & Wales, for birth cohorts 1951-1981 (at 5-year intervals) for Scotland, and for birth cohorts 1960-1986 for Northern Ireland. Data on percentage who breastfed initially was available for birth cohorts 1944-1980/81 (at around 5-year intervals though this varied depending on median year at first birth) for all UK countries, and earlier data was derived from publications describing the general state of UK breastfeeding in the early 20^th^ century. Data on percentage parous by age 45 (defined as percentage not childless by age 45 per cohort) was available for birth cohorts 1920-1970 for England & Wales, for birth cohorts 1930-1955 (at 5-year intervals) for Scotland, and for birth cohorts 1940-1965 (at 5-year intervals) for Northern Ireland.

Missing data in the middle of the series (e.g. where data were available in 5-year intervals) were imputed by assuming linear change between the bookending datapoints. Missing data at either end of the series were typically imputed by applying the average England & Wales versus Scotland/Northern Ireland ratio from existing datapoints, to England & Wales data for the missing datapoints (this was done for Scotland and Northern Ireland percentage parous, and Northern Ireland median year of first birth). Missing data at either end of the series for Scotland median year of first birth were replaced with England & Wales data because the ratios in the existing datapoints were inconsistent. Missing data at the end of the series for England & Wales percentage parous and for all countries’ percentage initially breastfeeding was replaced with the value at the end of the existing datapoints.

*Physical activity*

Prevalence of physical activity was provided as days per week on which at least 30 minutes of moderate physical activity was completed but the RRs were defined as metabolic-equivalent hours (MET-hours) per week. Days per week were converted to MET-hours per week assuming one hour of moderate activity is equal to four MET-hours, as defined in the source of the physical activity RRs and by the World Health Organization.^[[90]](#endnote-90)^ Using this conversion at least 5 days of 30+ minutes activity were required to exceed the reference category in the RR source (600 MET-minutes per week). It was not possible to identify people achieving 600+ MET-minutes in less than 5 days (e.g. 1 hour of moderate physical activity on 3 days per week).

Scotland data on physical activity were collected only in 2003 and 2008, so 2005 data were imputed assuming a linear trend between those two survey years.

*Postmenopausal hormones*

Prevalence of postmenopausal hormone use was provided as ever-use or current use, but the RRs were defined as current use or past use. Past use was calculated as the proportion who have ever used these products minus the proportion currently using them. Prevalence data did not specify which hormonal preparation was used (e.g. oestrogen-progestogen or oestrogen-only) so RRs for all preparations combined were used. Data were available only for England, Scotland and Wales so the averages of these countries were used for the Northern Ireland figures. Data on use of postmenopausal hormones were collected only for women up to age 74, so to impute figures for women aged 75+, ratios of use in women aged 65-74 versus women aged 75+ were calculated from a more recent survey,^[[91]](#endnote-91)^ and applied to the figures for women aged 65-74.

Postmenopausal hormones are associated with increased risk of some cancer types and decreased risk of others. As the outcome of interest in this project is attributable cases only, the cases theoretically avoided by use of postmenopausal hormones are not reported here. However as in the original UK attributable cancers project, it is likely that the net effect of postmenopausal use on cancer incidence in the UK is very small.

*Oral contraceptives*

Data on oral contraceptive use were not available for Northern Ireland so Republic of Ireland data were used as they were considered more representative of Northern Ireland than a GB average would be, given differences around contraception and abortion between Great Britain and Northern Ireland.^[[92]](#endnote-92)^ Data on use of oral contraceptives were collected only for women up to age 74, so to impute figures for women aged 75+, ratios of use in women aged 65-74 versus women aged 75+ were calculated from a more recent survey,83 and applied to the figures for women aged 65-74.

Oral contraceptives are also associated with increased risk of some cancer types and decreased risk of others, the cases theoretically avoided by their use are not reported here, and it is likely that the net effect of their use is very small.

# References for Supplementary Material

1. Gandini S, Botteri E, Iodice S, Boniol M, Lowenfels AB, Maisonneuve P, Boyle P (2008) Tobacco smoking and cancer: a meta-analysis. Int J Cancer 122(1): 155 – 64, DOI: 10.1002/ijc.23033 [↑](#endnote-ref-1)
2. Maasland DH, van den Brandt PA, Kremer B, Goldbohm RA, Schouten LJ (2014) Alcohol consumption, cigarette smoking and the risk of subtypes of head-neck cancer: results from the Netherlands Cohort Study. BMC Cancer 14: 187, DOI: 10.1186/1471-2407-14-187. [↑](#endnote-ref-2)
3. Tramacere I, La Vecchia C, Negri E (2011) Tobacco smoking and esophageal and gastric cardia adenocarcinoma: a meta-analysis. Epidemiology 22(3): 344 – 349, DOI: 10.1097/EDE.0b013e31821092cd. [↑](#endnote-ref-3)
4. Prabhu A1, Obi KO, Rubenstein JH (2013) Systematic review with meta-analysis: race-specific effects of alcohol and tobacco on the risk of oesophageal squamous cell carcinoma. Aliment Pharmacol Ther 38(10): 1145 – 1155, DOI: 10.1111/apt.12514 [↑](#endnote-ref-4)
5. Pandeya N, Williams GM, Sadhegi S, Green AC, Webb PM, Whiteman DC (2008) Associations of duration, intensity, and quantity of smoking with adenocarcinoma and squamous cell carcinoma of the esophagus. Am J Epidemiol 168(1): 105 – 114, DOI: 10.1093/aje/kwn091 [↑](#endnote-ref-5)
6. Ladeiras-Lopes R, Pereira AK, Nogueira A, Pinheiro-Torres T, Pinto I, Santos-Pereira R, Lunet N (2008) Smoking and gastric cancer: systematic review and meta-analysis of cohort studies. Cancer Causes Control 19(7): 689 – 701, DOI: 10.1007/s10552-008-9132-y [↑](#endnote-ref-6)
7. Lee YC, Cohet C, Yang YC, Stayner L, Hashibe M, Straif K (2009) Meta-analysis of epidemiologic studies on cigarette smoking and liver cancer. Int J Epidemiol 38(6): 1497 – 1511, DOI: 10.1093/ije/dyp280 [↑](#endnote-ref-7)
8. Bosetti C, Lucenteforte E, Silverman DT, Petersen G, Bracci PM, Ji BT, Negri E, Li D, Risch HA, Olson SH, Gallinger S, Miller AB, Bueno-de-Mesquita HB, Talamini R, Polesel J, Ghadirian P, Baghurst PA, Zatonski W, Fontham E, Bamlet WR, Holly EA, Bertuccio P, Gao YT, Hassan M, Yu H, Kurtz RC, Cotterchio M, Su J, Maisonneuve P, Duell EJ, Boffetta P, La Vecchia C (2012) Cigarette smoking and pancreatic cancer: an analysis from the International Pancreatic Cancer Case-Control Consortium (Panc4). Ann Oncol 23(7): 1880 – 1888, DOI: 10.1093/annonc/mdr541 [↑](#endnote-ref-8)
9. Cheng J, Chen Y, Wang X, Wang J, Yan Z, Gong G, Li G, Li C (2015) Meta-analysis of prospective cohort studies of cigarette smoking and the incidence of colon and rectal cancers. Eur J Cancer Prev 24(1): 6 – 15, DOI: 10.1097/CEJ.0000000000000011 [↑](#endnote-ref-9)
10. Collaborative Group on Epidemiological Studies of Ovarian Cancer, Beral V, Gaitskell K, Hermon C, Moser K, Reeves G, Peto R (2012) Ovarian cancer and smoking: individual participant meta-analysis including 28,114 women with ovarian cancer from 51 epidemiological studies. Lancet Oncol 13(9): 946 – 956, DOI: 10.1016/S1470-2045(12)70322-4 [↑](#endnote-ref-10)
11. van Osch FH, Jochems SH, van Schooten FJ, Bryan RT, Zeegers MP (2016) Quantified relations between exposure to tobacco smoking and bladder cancer risk: a meta-analysis of 89 observational studies. Int J Epidemiol 45(3): 857 – 870, DOI: 10.1093/ije/dyw044 [↑](#endnote-ref-11)
12. Cumberbatch MG, Rota M, Catto JW, La Vecchia C (2016) The Role of Tobacco Smoke in Bladder and Kidney Carcinogenesis: A Comparison of Exposures and Meta-analysis of Incidence and Mortality Risks. Eur Urol 70(3): 458 – 466, DOI: 10.1016/j.eururo.2015.06.042 [↑](#endnote-ref-12)
13. Fircanis S, Merriam P, Khan N, Castillo JJ (2014) The relation between cigarette smoking and risk of acute myeloid leukemia: an updated meta-analysis of epidemiological studies. Am J Hematol 89(8): E125 – E132, DOI:10.1002/ajh.23744 [↑](#endnote-ref-13)
14. Kim CH, Lee YC, Hung RJ, McNallan SR, Cote ML, Lim WY, Chang SC, Kim JH, Ugolini D, Chen Y, Liloglou T, Andrew AS, Onega T, Duell EJ, Field JK, Lazarus P, Le Marchand L, Neri M, Vineis P, Kiyohara C, Hong YC, Morgenstern H, Matsuo K, Tajima K, Christiani DC, McLaughlin JR, Bencko V, Holcatova I, Boffetta P, Brennan P, Fabianova E, Foretova L, Janout V, Lissowska J, Mates D, Rudnai P, Szeszenia-Dabrowska N, Mukeria A, Zaridze D, Seow A, Schwartz AG, Yang P, Zhang ZF (2014) Exposure to secondhand tobacco smoke and lung cancer by histological type: a pooled analysis of the International Lung Cancer Consortium (ILCCO). Int J Cancer 135(8): 1918 – 1930, DOI: 10.1002/ijc.28835 [↑](#endnote-ref-14)
15. Zuo JJ, Tao ZZ, Chen C, Hu ZW, Xu YX, Zheng AY, Guo Y (2017) Characteristics of cigarette smoking without alcohol consumption and laryngeal cancer: overall and time-risk relation. A meta-analysis of observational studies. Eur Arch Otorhinolaryngol 274(3): 1617 – 1631, DOI: 10.1007/s00405-016-4390-x [↑](#endnote-ref-15)
16. Munsell MF, Sprague BL, Berry DA, Chisholm G, Trentham-Dietz A (2014) Body mass index and breast cancer risk according to postmenopausal estrogen-progestin use and hormone receptor status. Epidemiol Rev 36: 114 – 136, DOI: 10.1093/epirev/mxt010. [↑](#endnote-ref-16)
17. Xue K, Li FF, Chen YW, Zhou YH, He J (2017) Body mass index and the risk of cancer in women compared with men: a meta-analysis of prospective cohort studies. Eur J Cancer Prev 26(1): 94 – 105, DOI: 10.1097/CEJ.0000000000000231 [↑](#endnote-ref-17)
18. Turati F, Tramacere I, La Vecchia C, Negri E (2013) A meta-analysis of body mass index and esophageal and gastric cardia adenocarcinoma. Ann Oncol 24(3): 609 – 617, DOI: 10.1093/annonc/mds244 [↑](#endnote-ref-18)
19. Wang F, Xu Y (2014) Body mass index and risk of renal cell cancer: a dose-response meta-analysis of published cohort studies. Int J Cancer 135(7):1673 – 1686, DOI: 10.1002/ijc.28813 [↑](#endnote-ref-19)
20. Jenabi E, Poorolajal J (2015)The effect of body mass index on endometrial cancer: a meta-analysis. Public Health 129(7): 872 – 880, DOI:: 10.1016/j.puhe.2015.04.017 [↑](#endnote-ref-20)
21. Aune D, Greenwood DC, Chan DS, Vieira R, Vieira AR, Navarro Rosenblatt DA, Cade JE, Burley VJ, Norat T (2012) Body mass index, abdominal fatness and pancreatic cancer risk: a systematic review and non-linear dose-response meta-analysis of prospective studies. Ann Oncol 23(4): 843 – 852, DOI: 10.1093/annonc/mdr398 [↑](#endnote-ref-21)
22. Aune D, Navarro Rosenblatt DA, Chan DS, Abar L, Vingeliene S, Vieira AR, Greenwood DC, Norat T (2015) Anthropometric factors and ovarian cancer risk: a systematic review and nonlinear dose-response meta-analysis of prospective studies. Int J Cancer 136(8): 1888 – 1898 DOI: 10.1002/ijc.29207 [↑](#endnote-ref-22)
23. Chen Y, Wang X, Wang J, Yan Z, Luo J (2012) Excess body weight and the risk of primary liver cancer: an updated meta-analysis of prospective studies. Eur J Cancer 48(14): 2137 – 2145, DOIi: 10.1016/j.ejca.2012.02.063 [↑](#endnote-ref-23)
24. Lin XJ, Wang CP, Liu XD, Yan KK, Li S, Bao HH, Zhao LY, Liu X (2014) Body mass index and risk of gastric cancer: a meta-analysis. Jpn J Clin Oncol 44(9): 783 – 791, DOI: 10.1093/jjco/hyu082 [↑](#endnote-ref-24)
25. Sergentanis TN, Tsivgoulis G, Perlepe C, Ntanasis-Stathopoulos I, Tzanninis IG, Sergentanis IN, Psaltopoulou T (2015) Obesity and Risk for Brain/CNS Tumors, Gliomas and Meningiomas: A Meta-Analysis. PLoS One 10(9): e0136974, DOI: 10.1371/journal.pone.0136974 [↑](#endnote-ref-25)
26. Schmid D, Ricci C, Behrens G, Leitzmann MF (2015) Adiposity and risk of thyroid cancer: a systematic review and meta-analysis. Obes Rev 16(12): 1042 – 1054, DOI: 10.1111/obr.12321 [↑](#endnote-ref-26)
27. Wallin A, Larsson SC (2011) Body mass index and risk of multiple myeloma: a meta-analysis of prospective studies. Eur J Cancer 47(11): 1606 – 1615, DOI: 10.1016/j.ejca.2011.01.020 [↑](#endnote-ref-27)
28. Discacciati A, Orsini N, Wolk A (2012) Body mass index and incidence of localized and advanced prostate cancer--a dose-response meta-analysis of prospective studies. Ann Oncol 23(7): 1665 – 1671, DOI: 10.1093/annonc/mdr603 [↑](#endnote-ref-28)
29. Parkin DM (2011) 11. Cancers attributable to infection in the UK in 2010. Brit J Cancer 105 (S2): S49 - S56, DOI: 10.1038/bjc.2011.484 [↑](#endnote-ref-29)
30. Cho LY, Yang JJ, Ko KP, Park B, Shin A, Lim MK, Oh JK, Park S, Kim YJ, Shin HR, Yoo KY, Park SK (2011) Coinfection of hepatitis B and C viruses and risk of hepatocellular carcinoma: systematic review and meta-analysis. Int J Cancer 128(1): 176 – 184, DOI: 10.1002/ijc.25321 [↑](#endnote-ref-30)
31. Pozzato G, Mazzaro C, Dal Maso L, Mauro E, Zorat F, Moratelli G, Bulian P, Serraino D, Gattei V (2016) Hepatitis C virus and non-Hodgkin's lymphomas: Meta-analysis of epidemiology data and therapy options. World J Hepatol 8(2): 107 – 116, DOI: 10.4254/wjh.v8.i2.107 [↑](#endnote-ref-31)
32. Carreira H, Coutinho F, Carrilho C, Lunet N (2013) HIV and HPV infections and ocular surface squamous neoplasia: systematic review and meta-analysis. Br J Cancer 109(7): 1981 – 1988, DOI: 10.1038/bjc.2013.539 [↑](#endnote-ref-32)
33. Gibson TM, Morton LM, Shiels MS, Clarke CA, Engels EA (2014) Risk of non-Hodgkin lymphoma subtypes in HIV-infected people during the HAART era: a population-based study. AIDS 28(15): 2313 – 2318, DOI: 10.1097/QAD.0000000000000428 [↑](#endnote-ref-33)
34. Bagnardi V, Rota M, Botteri E, Tramacere I, Islami F, Fedirko V, Scotti L, Jenab M, Turati F, Pasquali E, Pelucchi C, Galeone C, Bellocco R, Negri E, Corrao G, Boffetta P, La Vecchia C (2015) Alcohol consumption and site-specific cancer risk: a comprehensive dose-response meta-analysis. Br J Cancer 112(3): 580 – 593, DOI: 10.1038/bjc.2014.579 [↑](#endnote-ref-34)
35. Parkin DM, Boyd L (2011) 6. Cancers attributable to dietary factors in the UK in 2010. III. Low consumption of fibre. Br J Cancer 105 Suppl 2: S27 – 30, DOI: 10.1038/bjc.2011.479 [↑](#endnote-ref-35)
36. Health Protection Agency (2011) Risk of solid cancers following radiation exposure: Estimates for the UK population. Report of the independent advisory group on ionising radiation. Health Protection Agency: London [↑](#endnote-ref-36)
37. United Nations Scientific Committee on the Effects of Atomic Radiation (2006) UNSCEAR 2006 Report to the General Assembly, with scientific annexes. United Nations: Geneva [↑](#endnote-ref-37)
38. Chan DS, Lau R, Aune D, Vieira R, Greenwood DC, Kampman E, Norat T (2011) Red and processed meat and colorectal cancer incidence: meta-analysis of prospective studies. PLoS One 6(6): e20456, DOI: 10.1371/journal.pone.0020456 [↑](#endnote-ref-38)
39. Hamra GB, Guha N, Cohen A, Laden F, Raaschou-Nielsen O, Samet JM, Vineis P, Forastiere F, Saldiva P, Yorifuji T, Loomis D (2014) Outdoor particulate matter exposure and lung cancer: a systematic review and meta-analysis. Environ Health Perspect 122(9): 906 – 911, DOI: 10.1289/ehp.1408092 [↑](#endnote-ref-39)
40. Chowdhury R, Sinha B, Sankar MJ, Taneja S, Bhandari N, Rollins N, Bahl R, Martines J (2015) Breastfeeding and maternal health outcomes: a systematic review and meta-analysis. Acta Paediatr 104(467): 96 – 113, DOI: 10.1111/apa.13102 [↑](#endnote-ref-40)
41. Kyu HH, Bachman VF, Alexander LT, Mumford JE, Afshin A, Estep K, Veerman JL, Delwiche K, Iannarone ML, Moyer ML, Cercy K, Vos T, Murray CJL, Forouzanfar MH (2016) Physical Activity and Risk of breast cancer, colon cancer, diabetes, ischemic heart disease, and ischemic stroke events: systematic review and dose-response meta-analysis for the Global Burden of Disease Study 2013. BMJ: 354, DOI: 10.1136/bmj.i3857 [↑](#endnote-ref-41)
42. Beral V, Million Women Study Collaborators (2003) Breast cancer and hormone-replacement therapy in the Million Women Study. Lancet 362(9382):419 - 427 [↑](#endnote-ref-42)
43. Collaborative Group On Epidemiological Studies Of Ovarian Cancer, Beral V, Gaitskell K, Hermon C, Moser K, Reeves G, Peto R (2015) Menopausal hormone use and ovarian cancer risk: individual participant meta-analysis of 52 epidemiological studies. Lancet 385(9980): 1835 – 1842, DOI: 10.1016/S0140-6736(14)61687-1 [↑](#endnote-ref-43)
44. Gierisch JM, Coeytaux RR, Urrutia RP, Havrilesky LJ, Moorman PG, Lowery WJ, Dinan M, McBroom AJ, Hasselblad V, Sanders GD, Myers ER (2013) Oral contraceptive use and risk of breast, cervical, colorectal, and endometrial cancers: a systematic review. Cancer Epidemiol Biomarkers Prev 22(11): 1931 – 1943, DOI: 10.1158/1055-9965.EPI-13-0298 [↑](#endnote-ref-44)
45. International Collaboration of Epidemiological Studies of Cervical Cancer, Appleby P, Beral V, Berrington de González A, Colin D, Franceschi S, Goodhill A, Green J, Peto J, Plummer M, Sweetland S (2007) Cervical cancer and hormonal contraceptives: collaborative reanalysis of individual data for 16,573 women with cervical cancer and 35,509 women without cervical cancer from 24 epidemiological studies. Lancet 370(9599): 1609 – 1621, DOI: 10.1016/S0140-6736(07)61684-5 [↑](#endnote-ref-45)
46. NHS Digital (2016) Health Survey for England, 2015: Trend tables – Adult tables. Available from <http://www.content.digital.nhs.uk/catalogue/PUB22616>. Accessed October 2017. [↑](#endnote-ref-46)
47. Scottish Government (2016) Scottish Health Survey 2015 trend tables. Available from <http://www.gov.scot/Topics/Statistics/Browse/Health/scottish-health-survey/Publications>. Accessed October 2017. [↑](#endnote-ref-47)
48. National Assembly for Wales (2011) Welsh Health Survey, 1998. [data collection]. 2nd Edition. UK Data Service. SN: 4176, <http://doi.org/10.5255/UKDA-SN-4176-1> [↑](#endnote-ref-48)
49. Northern Ireland Statistics and Research Agency (2017) Continuous Household Survey Results: 9. Smoking. Available from <http://www.csu.nisra.gov.uk/survey.asp140.htm>. Accessed October 2017 [↑](#endnote-ref-49)
50. Northern Ireland Statistics and Research Agency. Northern Ireland Health and Social Wellbeing Survey 2005/06. Topline results – adult obesity. Available from <http://www.csu.nisra.gov.uk/adult%20obesity.pdf>. Accessed October 2017. [↑](#endnote-ref-50)
51. Office for National Statistics NOMIS Official Labour Market Statistics (2017) Workforce jobs by industry (SIC 2007) - seasonally adjusted (Sep 1981 to Jun 2017). Available from <https://www.nomisweb.co.uk/query/select/getdatasetbytheme.asp?theme=31>. Accessed October 2017. [↑](#endnote-ref-51)
52. Vyse AJ, Gay NJ, Hesketh LM, Andrews NJ, Marshall B, Thomas HI, Morgan-Capner P, Miller E (2002) The burden of Helicobacter pylori infection in England and Wales. Epidemiol Infect 128(3): 411 - 417 [↑](#endnote-ref-52)
53. McDonagh TA, Woodward M, Morrison CE, McMurray JJ, Tunstall-Pedoe H, Lowe GD, McColl KE, Dargie HJ (1997) Helicobacter pylori infection and coronary heart disease in the North Glasgow MONICA population. Eur Heart J 18(8): 1257 - 1260 [↑](#endnote-ref-53)
54. Murray LJ, McCrum EE, Evans AE, Bamford KB. Epidemiology of Helicobacter pylori infection among 4742 randomly selected subjects from Northern Ireland. Int J Epidemiol 26(4): 880 - 887 [↑](#endnote-ref-54)
55. Hepatitis B Foundation UK (2007) Rising Curve: Chronic Hepatitis B Infection in the UK. Hepatitis B Foundation UK: London [↑](#endnote-ref-55)
56. Harris RJ, Ramsay M, Hope VD, Brant L, Hickman M, Foster GR, De Angelis D (2012) Hepatitis C prevalence in England remains low and varies by ethnicity: an updated evidence synthesis. Eur J Public Health 22(2): 187 – 192, DOI: 10.1093/eurpub/ckr083 [↑](#endnote-ref-56)
57. Northern Ireland Department of Health, Social Services and Public Safety (2007) Action plan for the prevention, management and control of hepatitis C in Northern Ireland. DHSSPSNI: Belfast [↑](#endnote-ref-57)
58. National Public Health Service for Wales (2006) Blood Borne Viral Hepatitis Action for Wales Research Programme – Developing the evidence base. Findings, Implications and Recommendations. NPHS Wales: Wales [↑](#endnote-ref-58)
59. Hutchinson SJ, Roy KM, Wadd S, Bird SM, Taylor A, Anderson E, Shaw L, Codere G, Goldberg DJ (2006) Hepatitis C virus infection in Scotland: epidemiological review and public health challenges. Scott Med J 51(2): 8 – 15, DOI: 10.1258/RSMSMJ.51.2.8 [↑](#endnote-ref-59)
60. The UK Collaborative Group for HIV and STI Surveillance (2006) A Complex Picture. HIV and other Sexually Transmitted Infections in the United Kingdom: 2006. Health Protection Agency, Centre for Infections: London [↑](#endnote-ref-60)
61. Yin Z, Brown AE, Hughes G, Nardone A, Gill ON, Delpech VC & contributor (2014). HIV in the United Kingdom 2014 Report: data to end 2013. PHE: London [↑](#endnote-ref-61)
62. Office for National Statistics, Social and Vital Statistics Division (2007) General Household Survey, 2005. [data collection]. 2nd Edition. UK Data Service. SN: 5640, <http://doi.org/10.5255/UKDA-SN-5640-1> [↑](#endnote-ref-62)
63. Public Health England Centre for Radiation, Chemical and Environmental Hazards (2017). Personal communication January 2017. [↑](#endnote-ref-63)
64. Office for National Statistics and Medical Research Council Human Nutrition Research (2004) The National Diet & Nutrition Survey: adults aged 19 to 64 years. Summary Report. TSO: London [↑](#endnote-ref-64)
65. Food Standards Agency. National Diet and Nutrition Survey Rolling Programme (NDNS RP)

    Results from Years 1-4 (combined) for Scotland (2008/09-2011/12). Chapter 8: Tables. Available from <http://www.foodstandards.gov.scot/national-diet-and-nutrition-survey-rolling-programme-results-years-1-4-combined-scotland-200809>. Accessed October 2017. [↑](#endnote-ref-65)
66. Welsh Government. National Diet and Nutrition Survey Rolling Programme (NDNS) - Results from Years 2-5 (combined) for Wales (2009/10-2012/13). Chapter 8: Detailed age breakdowns for young people and adults in Wales for key nutrients and disaggregated foods and comparisons with the UK as a whole. Available from <http://gov.wales/statistics-and-research/national-diet-nutrition-survey-rolling-programme/?lang=en>. Accessed October 2017. [↑](#endnote-ref-66)
67. Food Standards Agency. National Diet and Nutrition Survey Rolling Programme (NDNS RP)

    Results from Years 1-4 (combined) for Northern Ireland (2008/09-2011/12). Chapter 8: Tables. Available from <https://www.food.gov.uk/northern-ireland/researchni/ndns-ni>. Accessed October 2017. [↑](#endnote-ref-67)
68. Public Health England (2014) Estimating local mortality burdens associated with particulate air pollution. PHE: Chilton [↑](#endnote-ref-68)
69. Office for National Statistics (2016) Childbearing for women born in different years. Available from <https://www.ons.gov.uk/peoplepopulationandcommunity/birthsdeathsandmarriages/conceptionandfertilityrates/datasets/childbearingforwomenbornindifferentyearsreferencetable>. Accessed October 2017. [↑](#endnote-ref-69)
70. Office for National Statistics (2004) Population Trends. 117, p34. [↑](#endnote-ref-70)
71. National Records of Scotland (2016) Vital Events Reference Tables 2015. Section 3: Births. Available from <https://www.nrscotland.gov.uk/statistics-and-data/statistics/statistics-by-theme/vital-events/general-publications/vital-events-reference-tables/2015/section-3-births>. Accessed October 2017. [↑](#endnote-ref-71)
72. OECD Family Database (2015) The structure of families. Fertility indicators. SF2.5 Childlessness [data for Ireland]. Available from <http://www.oecd.org/social/family/database.htm>. Accessed October 2017. [↑](#endnote-ref-72)
73. Northern Ireland Statistics and Research Agency (2017) Monthly births. Available from <http://www.nisra.gov.uk/demography/default.asp8.htm>. Accessed October 2017. [↑](#endnote-ref-73)
74. McAndrew F, Thompson J, Fellows L, Large A, Speed M, Renfrew MJ (2012) Infant Feeding Survey 2010. The Health and Social Care Information Centre: London [↑](#endnote-ref-74)
75. Lawrence W Jr, Miller DG, Isaacs M, Whitmore W (1965) Nutrition in Pregnancy and Lactation. Report of a WHO Expert Committee. World Health Organ Tech Rep Ser 302: 1 - 54 [↑](#endnote-ref-75)
76. Bryder L (2005) Breastfeeding and health professionals in Britain, New Zealand and the United States, 1900--1970. Med Hist 49(2): 179 - 196 [↑](#endnote-ref-76)
77. Johnson A, London School of Hygiene and Tropical Medicine Centre for Sexual and Reproductive Health Research, NatCen Social Research, Mercer C (2017) National Survey of Sexual Attitudes and Lifestyles, 2010-2012. [data collection]. 2nd Edition. UK Data Service. SN: 7799, <http://doi.org/10.5255/UKDA-SN-7799-2> [↑](#endnote-ref-77)
78. Crisis Pregnancy Programme (2012) Irish Contraception and Crisis Pregnancy Study 2010 (ICCP-2010). A Survey of the General Population. HSE Crisis Pregnancy Programme: Dublin [↑](#endnote-ref-78)
79. Scottish Government (2005) Smoking, Health and Social Care (Scotland) Act 2005. TSO: Scotland [↑](#endnote-ref-79)
80. Armstrong BK, Kricker A (1993) How much melanoma is caused by sun exposure? Melanoma Res 3(6): 395 - 401. [↑](#endnote-ref-80)
81. Parkin DM (2011) 14. Cancers attributable to occupational exposures in the UK in 2010. Br J Cancer 105 Suppl 2: S70 – S72, DOI: 10.1038/bjc.2011.487 [↑](#endnote-ref-81)
82. National Cancer Registration and Analysis Service (2010). The Importance of Skin Cancer Registration. NCIN Data Briefing. NCRAS: London [↑](#endnote-ref-82)
83. Internation Agency for Research on Cancer (2010). IARC Monographs on the Evaluation of Carcinogenic Risks to Humans. Volume 98 (2010). Painting, Firefighting, and Shiftwork. IARC: Lyon [↑](#endnote-ref-83)
84. National Institute for Health and Care Excellence (2013) Hepatitis B and C testing: people at risk of infection. Public health guideline [PH43]. NICE: London [↑](#endnote-ref-84)
85. Drinkaware (2017) What is an alcohol unit? Available from <https://www.drinkaware.co.uk/alcohol-facts/alcoholic-drinks-units/what-is-an-alcohol-unit/>. Accessed October 2017. [↑](#endnote-ref-85)
86. Public Health England and Food Standards Agency (2014) NDNS: Results from Years 1-4 (combined). Available from <https://www.gov.uk/government/statistics/national-diet-and-nutrition-survey-results-from-years-1-to-4-combined-of-the-rolling-programme-for-2008-and-2009-to-2011-and-2012>. Accessed October 2017. [↑](#endnote-ref-86)
87. NRPB (1987). Exposure to Radon Daughters in Dwellings. Health Protection Agency: Chilton [↑](#endnote-ref-87)
88. Parkin DM, Darby SC (2011) 12. Cancers in 2010 attributable to ionising radiation exposure in the UK. Br J Cancer 105 Suppl 2: S57 - S65, DOI: 10.1038/bjc.2011.485 [↑](#endnote-ref-88)
89. Macmillan Cancer Support and National Cancer Registration and Analysis Service (2017) UK Cancer Prevalence Project. Updated UK Complete Cancer Prevalence for 2013 Workbook for UK nations and UK combined. Available from: <http://www.ncin.org.uk/about_ncin/segmentation>. Accessed October 2017. [↑](#endnote-ref-89)
90. World Health Organization (2017) What is Moderate-intensity and Vigorous-intensity Physical Activity? Available from <http://www.who.int/dietphysicalactivity/physical_activity_intensity/en/>. Accessed October 2017 [↑](#endnote-ref-90)
91. National Centre for Social Research, University College London Department of Epidemiology and Public Health (2011) Health Survey for England, 2006. [data collection]. 4th Edition. UK Data Service. SN: 5809, <http://doi.org/10.5255/UKDA-SN-5809-1> [↑](#endnote-ref-91)
92. Marie Stopes UK. Is abortion legal in Ireland? Available from: <https://www.mariestopes.ie/abortion-care/is-abortion-legal-in-ireland/>. Accessed October 2017. [↑](#endnote-ref-92)
